# A progressive ODE model of seasonal allergic rhinitis: from core Th2 axis to 21-dimensional multi- pathway integration with seasonal dynamics and directional assessment

**DOI:** 10.3389/fimmu.2026.1971520

**Published:** 2026-10-29

**Authors:** Qiang Zhang, Houyang Hu, Yi Yue, Wenwen Qi, Wenqing Zhu, Xinran Liu, Xuanchen Zhou

**Affiliations:** Department of Otorhinolaryngology Head and Neck Surgery, Shandong Provincial Hospital Affiliated to Shandong First Medical University, Jinan, China

**Keywords:** dupilumab, nasal allergen challenge, ordinary differential equation model, seasonal allergic rhinitis, Sobol sensitivity analysis, Th17/Treg imbalance, TLR4/NF-κB signaling, virtual patient cohort

## Abstract

**Background:**

Seasonal allergic rhinitis (SAR) affects approximately 400 million individuals worldwide, yet a quantitative framework integrating the complete immunological cascade from allergen exposure to clinical symptoms is lacking. Individual pathways (Th2/IgE, TLR4/NF-κB, Th17/Treg) have been characterized in isolation, but their interactions and relative contributions to symptom generation remain poorly understood.

**Methods:**

We constructed a progressively extended ordinary differential equation (ODE) model evolving from a 10-dimensional core Th2/IgE/mast cell/eosinophil axis to a 21-dimensional system integrating TLR4/MyD88/NF-κB innate immunity and Th17/Treg imbalance with seasonal priming and tolerance dynamics. The model was calibrated against published cytokine data (Leaker 2016, Linton 2025) and validated against clinical trial drug efficacy (Corren 2021, Bauer 2023). Sobol global sensitivity analysis (5,888 samples, 21 parameters) was performed. A 2,000-patient virtual cohort was generated with lognormal parameter distributions (CV = 30%), and PCA-reduced K-means clustering (PC1–PC2 of six output metrics) identified virtual subtypes. Convergence was verified by doubling the base sample size to N = 512: all S1 and ST indices changed by less than 0.013, confirming adequate convergence at the original N = 256.

**Results:**

The core model reproduces the biphasic symptom profile (immediate peak 7.0 at 1.9h, late plateau 3.4 at 12-24h) and reveals a mechanistic dichotomy: the immediate peak is governed by the mast cell-mediator axis while total burden is controlled by the eosinophil-resolution axis. The 19-dimensional extension shows that TLR4 and Th17/Treg pathways primarily modulate the late-phase response (11.3% AUC reduction via IL-10 suppression). Sobol analysis identifies the IL-10/Treg axis as the 3rd-4th most important determinant of total symptom burden. The seasonal extension captures immunological priming (IL-5 + 46.1%). Three virtual subtypes map to published AR phenotypes. The model predicts that the Th17/Treg ratio shows a stronger directional association with late-phase severity than IgE, consistent with published clinical data.

**Conclusion:**

This progressive ODE model provides a quantitative framework for understanding SAR pathway interactions and generates five testable hypotheses for future experimental validation. The finding that 22% of simulated individuals in the virtual cohort exhibit a severe IL-17-high phenotype supports the hypothesis that Th17/Treg-targeted therapies may benefit a biologically defined subgroup of SAR patients, warranting validation in real patient cohorts.

## 1 Introduction

Seasonal allergic rhinitis (SAR) is a global health concern affecting approximately 400 million people, characterized by IgE-mediated type 2 immune responses to seasonal aeroallergens such as tree, grass, and weed pollens (1, 2). The pathophysiology of SAR involves a complex immunological cascade initiated by allergen penetration through the nasal epithelial barrier, triggering the release of epithelial-derived alarmins (TSLP, IL-25, IL-33) that activate innate and adaptive type 2 immune responses (3, 4).

The canonical SAR response is biphasic: an immediate phase (within minutes) driven by mast cell degranulation releasing histamine and leukotrienes, and a late phase (4–24 hours) characterized by eosinophil infiltration and sustained Th2 cytokine production (5, 6). While individual pathways have been extensively characterized, a quantitative mathematical framework integrating the complete cascade from allergen exposure to clinical symptom expression is needed to understand pathway interactions, identify rate-limiting steps, and predict therapeutic intervention outcomes.

Ordinary differential equation (ODE) models have been successfully applied to infectious disease dynamics and immune response modeling (7, 8), but their application to allergic rhinitis remains limited. Recent research has identified two additional pathways that significantly modulate SAR pathophysiology and are not captured by existing core Th2 models:

TLR4/MyD88/NF-κB innate immunity pathway: Pollen allergens directly activate TLR4/MD2 complexes on epithelial cells, triggering MyD88-dependent NF-κB signaling. This produces pro-inflammatory cytokines (TNF-α, IL-6, IL-1β) and amplifies alarmin (TSLP, IL-33) release. TLR4-deficient mice show eliminated IL-33 and Th2 responses, demonstrating the critical role of this pathway in initiating the adaptive cascade (9, 10).

Th17/Treg imbalance axis: In allergic rhinitis patients, Th17 cells are elevated while Treg cells are suppressed, with the Th17/Treg ratio correlating with disease severity (11, 12). IL-17 produced by Th17 cells amplifies Th2 responses, promotes IgE class switching, and recruits neutrophils. Conversely, Treg-derived IL-10 provides immunosuppression. Anti-IL-17 therapy has been shown to reduce Th2/Th17 responses and increase Treg, alleviating symptoms (13).

We adopt a progressive model-building strategy, starting with a 10-dimensional core model capturing the essential Th2/IgE/mast cell/eosinophil axis, then extending to 19 dimensions by incorporating TLR4/NF-κB and Th17/Treg pathways, validating against literature and clinical trial data, and finally extending to 21 dimensions with seasonal priming and tolerance dynamics. This approach allows systematic assessment of each pathway’s contribution and ensures that model complexity is justified at each stage.

The model is explicitly positioned as a hypothesis-generating tool. Rather than claiming clinical predictive accuracy, we derive five specific, experimentally testable predictions from the model’s structure and simulations. Each hypothesis includes the model prediction, a proposed validation approach, and the data required to test it. One hypothesis (H4: Th17/Treg ratio predicts late-phase severity) is examined against published clinical data within this work, though the synthetic cohort limits the strength of this assessment.

## 2 Materials and methods

### 2.1 Core 10-dimensional model

The initial model comprises 10 state (Equations 1–10, **Table 1**) variables representing the core SAR immunological cascade, organized into three tiers: upstream (allergen, alarmins), midstream (DC, Th2, cytokines, IgE, mast cells, mediators), and downstream (eosinophils, symptoms). The system is described by 10 coupled ODEs with 26 parameters (**Supplementary Table S1**; state variables are listed in **Supplementary Table S2**):

**Table 1:** Core model state variables and initial conditions.

Var	Description	Unit	IC	Biological basis
A	Allergen concentration	ng/mL	100	Single pollen exposure bolus
Al	Epithelial alarmins (TSLP+IL-33)	pg/mL	0	No pre-existing damage
DC	Activated dendritic cells	cells/µL	5	Baseline mucosal DC
Th2	Activated Th2 cells	cells/µL	2	Memory Th2 (sensitized)
Cyto	Th2 cytokines (IL-4/5/13)	pg/mL	0	No acute inflammation
IgE	Allergen-specific IgE	ng/mL	150	Pre-existing (sensitized)
MC	Mast cell activation fraction	0-1	0	Not activated
Med	Inflammatory mediators	ng/mL	0	Not released
Eos	Nasal eosinophils	cells/µL	10	Baseline few
Sym	Symptom score	0-10	0	Asymptomatic

(1) dA/dt=−k_A·A

(2) dAl/dt=k_Al1·A·(1−Al/Al_max)−k_Al2·Al

(3) dDC/dt=k_DC1·Al·(1−DC/DC_max)−k_DC2·DC

(4) dTh2/dt=k_T1·DC·(1−Th2/Th2_max)−k_T2·Th2

(5) dCyto/dt=k_C1·Th2−k_C2·Cyto

(6) dIgE/dt=k_IgE0+k_IgE1·Cyto−k_IgE2·IgE

(7) dMC/dt=k_MC1·A·IgE·(1−MC)−k_MC2·MC

(8) dMed/dt=k_Med1·MC−k_Med2·Med

(9) dEos/dt=k_Eos1·Cyto·(1−Eos/Eos_max)−k_Eos2·Eos

(10) dSym/dt=(k_S1·Med+k_S2·Eos)·(1−Sym/Sym_max)−k_S5·Sym

Saturation terms (1 - X/X_max) were incorporated for alarmin production, DC and Th2 activation, mast cell degranulation, eosinophil tissue capacity, and symptom scoring to ensure biological realism and bounded state variables. The mast cell activation term (Equation 7) models Fc-ϵRI cross-linking as the product of allergen and IgE concentrations, consistent with the mechanism of immediate-phase hypersensitivity. Note: The symptom-resolution rate parameter is denoted k_S5 throughout this manuscript for consistency; an earlier draft used k_S3 in Equation 10, which has been unified to k_S5.

### 2.2 Extended 19-dimensional model

The 19-dimensional model adds 9 state (Equations 11–19) variables across two new pathways. The TLR4 cascade is modeled as a four-step signaling amplifier: allergen binds TLR4 → recruits MyD88 → activates NF-κB → produces pro-inflammatory cytokines and recruits neutrophils. Cross-connections to the core axis include NF-κB-driven alarmin enhancement and DC activation. The complete list of 19 state variables is provided in **Supplementary Table S2**. The 31 new parameters associated with these pathways are listed in **Supplementary Table S3**.

(11) dTLR4/dt=k_TLR1·A·(1−TLR4)−k_TLR2·TLR4

(12) dMyD88/dt=k_MyD1·TLR4·(1−MyD88)−k_MyD2·MyD88

(13) dNFkB/dt=k_NF1·MyD88·(1−NFkB)−k_NF2·NFkB

(14) dProInf/dt=k_PI1·NFkB−k_PI2·ProInf

(15) dNeu/dt=(k_Neu1·ProInf+k_Neu1IL17·IL17)·(1−Neu/Neu_max)−k_Neu2·Neu

Th17 differentiation requires both DC antigen presentation and IL-6 (contained in ProInf). Treg expansion is DC-dependent but suppressed by Th17. The two cell populations mutually antagonize each other, creating an imbalance that drives chronic inflammation. IL-17 amplifies Th2 activation and neutrophil recruitment, while IL-10 suppresses DC activity and symptoms.

(16) dTh17/dt=k_Th17_1·DC·ProInf·(1−Th17/Th17_max)−k_Th17_2·Th17−k_Treg_inh·Treg

(17) dIL17/dt=k_IL17_1·Th17−k_IL17_2·IL17

(18) dTreg/dt=k_Treg_1·DC·(1−Treg/Treg_max)−k_Treg_2·Treg−k_Th17_inh·Th17

(19) dIL10/dt=k_IL10_1·Treg−k_IL10_2·IL10

Four core equations were modified to incorporate cross-pathway interactions: Equation 2 Alarmins: + k_AlNF · NFkB (NF-κB amplifies alarmin release); Equation 3 DC: + k_DCNF · NFkB - k_IL10_DC · IL10 · DC (NF-κB enhances, IL-10 suppresses); Equation 4 Th2: + k_T1IL17 · IL17 (IL-17 amplifies Th2 activation); Equation 10 Symptoms: + k_S3b·Neu + k_S4·ProInf - k_S_IL10·IL10 (neutrophils, pro-inflammatory, IL-10 suppression).

### 2.3 Seasonal 21-dimensional extension

Two new state variables were added to simulate an entire pollen season. Priming (P): Accumulates with repeated allergen exposure (rate k_Prime1 = 0.08/h), decays slowly (t_1/2_ ~ 6 days). Amplifies alarmin release (k_Al1) and DC activation (k_DC1) by a factor of (1 + 2.0×P), representing mucosal sensitization from repeated exposure. Tolerance (T): Slowly accumulates with cumulative exposure (rate k_Tol1 = 0.003/h), very slow decay (t_1/2_ ~ 60 days). Dampens Th2 differentiation (k_T1) and cytokine production (k_C1) by (1 - 0.5×T), representing Treg expansion and immune regulation.

The allergen input A(t) is driven by a realistic pollen season profile: a Gaussian seasonal envelope (peak at day 21, spread ±14 days) modulated by daily diurnal variation (peak at 2pm) and day-to-day stochastic variation. The 56-day simulation uses 1-hour time steps with Livermore Solver for Ordinary Differential Equations with automatic stiffness detectio(LSODA) integration. The priming and tolerance dynamics are governed by: (20) dP/dt = k_Prime1 · A · (1 - P) - k_Prime2 · P, where k_Prime1 = 0.08/h (priming accumulation rate) and k_Prime2 = 0.002/h (decay t½ ~ 6 days). P amplifies alarmin release and DC activation by factor (1 + 2.0·P). (21) dT/dt = k_Tol1 · A_cumulative · (1 - T) - k_Tol2 · T, where k_Tol1 = 0.003/h (tolerance accumulation rate), k_Tol2 = 0.0002/h (decay t½ ~ 60 days), and A_cumulative is the cumulative allergen exposure. T dampens Th2 differentiation and cytokine production by factor (1 - 0.5·T). Both P and T are bounded in [0, 1] by the saturation terms.

### 2.4 Parameterization and calibration

A mixed parameterization strategy was employed across three calibration stages. Stage 1 (Core model): 7 parameters (27%) were derived from published immunological literature (e.g., IgE half-life ~2.5 days, histamine clearance t_1/2_ ~10 min, DC tissue half-life ~6h); 14 parameters (54%) were estimated based on biological plausibility; 2 parameters (8%) were calibrated to reproduce clinically observed symptom scores; 1 parameter (4%) was calculated from steady-state constraints; 1 parameter (4%) was literature-adjusted, and 1 parameter (Sym_max = 10.0) was fixed. Stage 2 (Fold-change calibration): Six key parameters from the TLR4/MyD88/NF-κB and Th17/Treg pathways were calibrated against quantitative cytokine time-course data from two published Nasal Allergen Challenge (NAC) studies: Leaker et al. (2016) (14) (n=19, IL-5 peaked at 3.5-fold, IL-1β at 10.7-fold) and Baumann et al. (2013) (15) (n=6, IL-17 and eotaxin-3 correlated r=0.94). L-BFGS-B constrained optimization minimized the weighted sum of squared log-residuals, with parameters bounded at 0.3× to 3.0× their original values. The estimated parameter proportion decreased from 61% to 51%. Stage 3 (Absolute pg/mL calibration): Five scaling factors (scale_Cyto, scale_ProInf, scale_IL17, scale_IL10, scale_Eos) were introduced to map the dimensionless aggregate state variables (Cyto, ProInf, IL17, IL10) to absolute pg/mL units; Eos, already in cells/µL, was scaled separately. L-BFGS-B optimization minimized the weighted sum of squared residuals (normalized by literature SEM) between model and Linton et al. (2025) (16) at 0, 1, 6, and 24h. Two production rates (k_C1, k_PI1) were co-calibrated to improve the cytokine time-course shape. The model uses aggregated state variables for cytokines: Cyto represents the combined concentration of IL-4, IL-5, and IL-13, while ProInf represents TNF-α, IL-6, and IL-1β. Individual cytokine concentrations for calibration were obtained using fixed proportional splitting factors derived from Linton et al. (2025) baseline ratios: IL-5 = 0.35 × Cyto, IL-13 = 0.25 × Cyto, IL-4 = 0.40 × Cyto; TNF-α = 0.45 × ProInf, IL-6 = 0.35 × ProInf, IL-1β = 0.20 × ProInf. These ratios were held constant across all simulations. The final calibrated value of k_C1 used in all reported simulations is 0.8 (from Stage 3 absolute calibration, **Supplementary Table S9**); the Stage 2 fold-change value of 2.4 (**Supplementary Table S8**) was superseded by the Stage 3 re-calibration.

### 2.5 Sensitivity analysis

Two sensitivity analysis methods were employed sequentially. One-at-a-time (OAT): ± 10% parameter perturbation applied to all parameters of both models (26 parameters in the 10-dim model, 57 in the 19-dim model, ×2 directions each), measuring three output metrics: symptom peak (Sym_max), time-to-peak (t_peak), and area under the symptom curve (AUC_0-24h). Sobol global sensitivity analysis: Saltelli sampling with N = 256 base samples generated 5,888 parameter combinations across 21 key parameters selected based on OAT screening (all 12 core axis parameters + 4 TLR4 pathway parameters and 5 Th17/Treg axis parameters with the highest OAT sensitivity; parameters with negligible OAT sensitivity (<0.5% AUC change) were excluded to reduce computational cost) (12 core axis + 4 TLR4 pathway + 5 Th17/Treg axis), each varied over 0.5× to 2.0× baseline. Sobol indices were computed for Sym_max and AUC, capturing both first-order (S1: individual parameter effect) and total-order (ST: parameter effect including all interactions) sensitivity (17, 18).

### 2.6 Virtual cohort and AI methods

A virtual cohort of 2,000 patients was generated by sampling 56 model parameters (Sym_max was held fixed at 10.0 as it is a scoring scale definition rather than a biological parameter) and 6 initial conditions from lognormal distributions (CV = 30%) around their baseline values. The 19-dim ODE system was solved for each patient (2,000/2,000 successful, 4.1s with 4-core parallelization). Three AI methods were applied: (1) a multi-layer perceptron (MLP) surrogate model (128-64–32 hidden layers, ReLU activation, Adam optimizer) trained to predict six output metrics from 62 input features, evaluated using 5-fold cross-validation; (2) permutation importance analysis (10 repeats) for feature ranking; (3) PCA-reduced K-means clustering on the first two principal components of six standardized output metrics (PC1 32.9% + PC2 24.8% = 57.7% cumulative variance), with silhouette analysis identifying K = 3 as optimal (silhouette = 0.36). Cluster stability was assessed across 10 random seeds (ARI = 0.98, range 0.95–1.00) and via bootstrap resampling (80% subsampling, 100 iterations: ARI = 0.94 ± 0.04). The K = 3 solution was also compared with K = 2 (silhouette = 0.35), K = 4 (0.33), and K = 5 (0.35), confirming K = 3 as optimal (**Supplementary Table S21**).

### 2.7 Drug intervention simulations

Eight therapeutic interventions were simulated by modifying specific model parameters to reflect known pharmacological mechanisms. Four original drugs (antihistamine, omalizumab, dupilumab, tezepelumab) target the core axis. Four novel targets enabled by the extended pathway structure include anti-IL-17 (secukinumab, k_IL17_1 × 0.1), anti-TLR4 (k_TLR1 × 0.1), anti-TNF-α (k_PI1 × 0.2), and Treg enhancement (low-dose IL-2, k_Treg_1 × 3, Treg(0) × 2). Subtype-specific drug response was assessed by simulating each subtype’s representative patient (median parameters) under four drug interventions.

For intranasal corticosteroids (INCS), the model was expanded to seven biologically-motivated targets: alarmin release (k_Al1), Th2 cytokine production (k_C1), NF-κB activation (k_NF1), pro-inflammatory cytokine output (k_PI1), mast cell sensitization (k_MC1), mediator release (k_Med1), and eosinophil recruitment (k_Eos1). To avoid overfitting (7 parameters vs 2 clinical targets), six factors were fixed from published quantitative data: k_Al1 × 0.16 (84% TSLP suppression by fluticasone; Kato 2007 (19)), k_NF1 × 0.50 (49% NF-κB transrepression; Lidén 2003 (20)), k_PI1 × 0.25 (~75% IL-6/IL-8 reduction; Lidén 2003 (20), Escotte 2003 (21)), k_MC1 × 0.85 and k_Med1 × 0.85 (minimal direct mast cell inhibition; Schleimer 1983 (22)), and k_Eos1 × 0.35 (~65% eosinophil reduction; Wierzchowska 2023 (23)). Only k_C1 was optimized (× 0.105) against the IL-13 reduction target (Bauer 2023 (24)).

To test the robustness of drug-effect predictions across a range of blockade intensities, a parametric sweep was performed for anti-TLR4 (k_TLR1) and anti-IL-17 (k_IL17_1) at five blockade factors (×0.01, ×0.05, ×0.1, ×0.2, and ×0.5, where the factor represents the fraction of baseline activity retained; e.g., ×0.01 corresponds to 99% blockade). All pairwise combinations of anti-TLR4 and anti-IL-17 were simulated at each blockade level. Synergy was assessed using the Bliss independence model, which predicts the combined effect under the assumption of independent action: E_expected = E_A + E_B − E_A × E_B. A Bliss excess (E_observed − E_expected) > 0 indicates synergy, = 0 indicates additivity, and < 0 indicates antagonism (sub-additive effect). Full sweep results are provided in **Supplementary Table S19**, and the Bliss synergy analysis is provided in **Supplementary Table S20**.

### 2.8 Literature validation data sources

Eleven published studies provided validation data across multiple model components: Leaker et al. (2016) (14) and Baumann et al. (2013) (15) for fold-change cytokine calibration; Linton et al. (2025) (16) and Campion et al. (2023) (25) for absolute cytokine concentrations and late-phase responder phenotyping; Corren et al. (2021) (26) for dupilumab clinical trial efficacy (n=103, SCIT+dupilumab vs placebo, between-group difference of -33.9% in TNSS AUC 0-1h post-NAC); Bauer et al. (2023) (24) for intranasal corticosteroid efficacy (n=20, INCS vs placebo, between-group effect sizes: -93% IL-13 at 7h, -32% TNSS AUC 0-30min); de Weger et al. (2011) (27) for seasonal symptom-pollen dynamics (r=0.51-0.65, early-late difference -0.30 to -0.41); Orban et al. (2020) (28) for repetitive NAC priming (IL-5 increase p=0.01); Cai et al. (2024) (29) for miR-135a correlations with both TNSS and Treg/Th17 balance (all p<0.001), from which an indirect link between Th17/Treg imbalance and symptom severity can be inferred; Xiong et al. (2026) (12) for Th17/Treg imbalance characterization; Gu et al. (2017) (13) for anti-IL-17 therapy in AR mouse model.

### 2.9 Numerical methods

The ODE system was solved using https://docs.scipy.org/doc/scipy/reference/generated/scipy.integrate.solve_ivp.html with the LSODA method (adaptive step size, rtol=1e-8, atol=1e-10), suitable for mixed stiff/non-stiff systems. Acute simulations ran over t = (0, 24) hours with output intervals of 0.05h. The seasonal simulation ran over 56 days with 1-hour time steps. Virtual cohort simulations used 4-core parallelization via joblib.Parallel. All analyses were implemented in Python 3.11 using scipy, numpy, pandas, scikit-learn, and matplotlib. All state variables are constrained by saturation terms (1 - X/X_max) to remain biologically bounded: variables with carrying capacities (Al, DC, Th2, MC, Eos, Neu, Th17, Treg) remain in [0, X_max], while signaling fractions (TLR4, MyD88, NF-κB) are bounded in (0, 1). Under standard parameters, numerical verification confirmed that all state variables remained non-negative throughout the simulation. In the virtual cohort, however, the symptom score (Sym) reached a minimum of −0.48 under extreme IL-10 suppression (affecting < 3% of patients), because IL-10 suppression can exceed the symptom generation rate. Sym values were clamped to (0, 10) in post-processing. All downstream analyses (AUC calculation, K-means clustering, ROC analysis) were performed on these post-clamped values.

## 3 Results

### 3.1 Core model dynamics (10-dimensional)

The 10-dimensional core model (**Figure 1**) captures the SAR cascade from allergen exposure through alarmin release, DC activation, Th2 polarization, IgE-mediated mast cell degranulation, to eosinophil-driven late-phase inflammation. Following a single allergen bolus (A_0_ = 100 ng/mL), the model reproduces the characteristic biphasic response over 24 hours (**Figure 2**): allergen is 95% cleared within 2 h, mast cells activate rapidly (84% degranulation at 0.4 h), mediators peak at 0.9 h and clear quickly (t_1/2_ ~ 10 min), while eosinophils peak later at 174 cells/µL at 14 h. This reveals a clear mechanistic dichotomy: the mast cell–mediator axis drives the immediate phase, while the eosinophil axis sustains the late-phase burden. Full variable dynamics are summarized in **Supplementary Table S4**.

**Figure 1:**
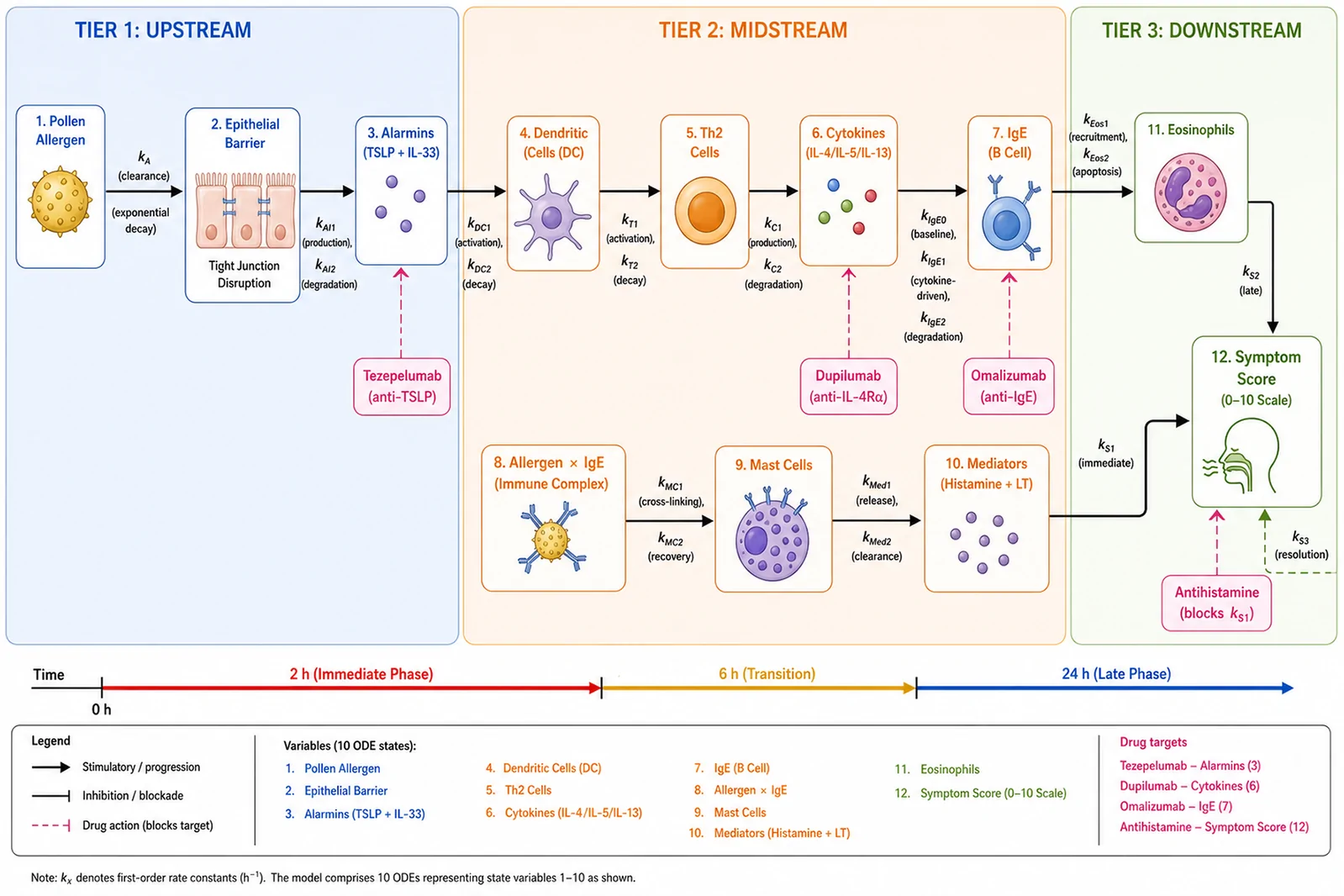
Schematic of the SAR core pathway ODE model. The 10-state-variable cascade flows from allergen exposure (left) through epithelial, innate, and adaptive immune activation to clinical symptoms (right). Drug intervention targets are annotated with dashed arrows.

**Figure 2:**
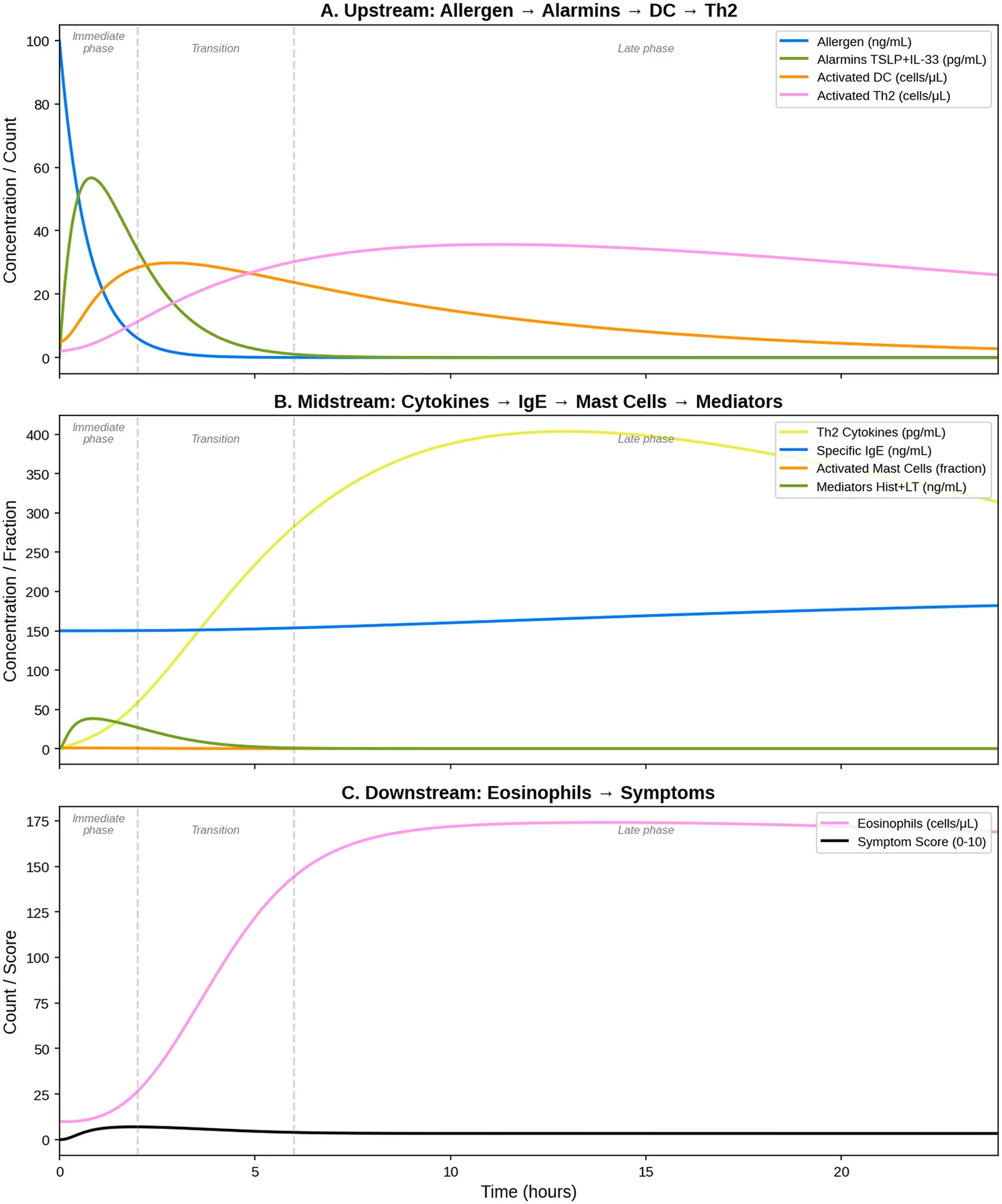
Temporal dynamics of all 10 model variables over 0–24 hours. **(A)** Allergen clearance and alarmin release. **(B)** DC and Th2 cell kinetics. **(C)** Cytokine production and IgE dynamics. **(D)** Mast cell activation and mediator release. **(E)** Eosinophil infiltration. **(F)** Biphasic symptom score. Red shading: immediate phase (0-2h); blue shading: late phase (6-24h).

The biphasic symptom structure is further illustrated in **Figure 3**, showing an immediate peak (7.0 at 1.9 h), inter-phase dip, and late-phase plateau (3.4 at 12 h). The sharp decline from peak to dip reflects rapid mediator clearance, while the sustained plateau reflects slower eosinophil kinetics.

**Figure 3:**
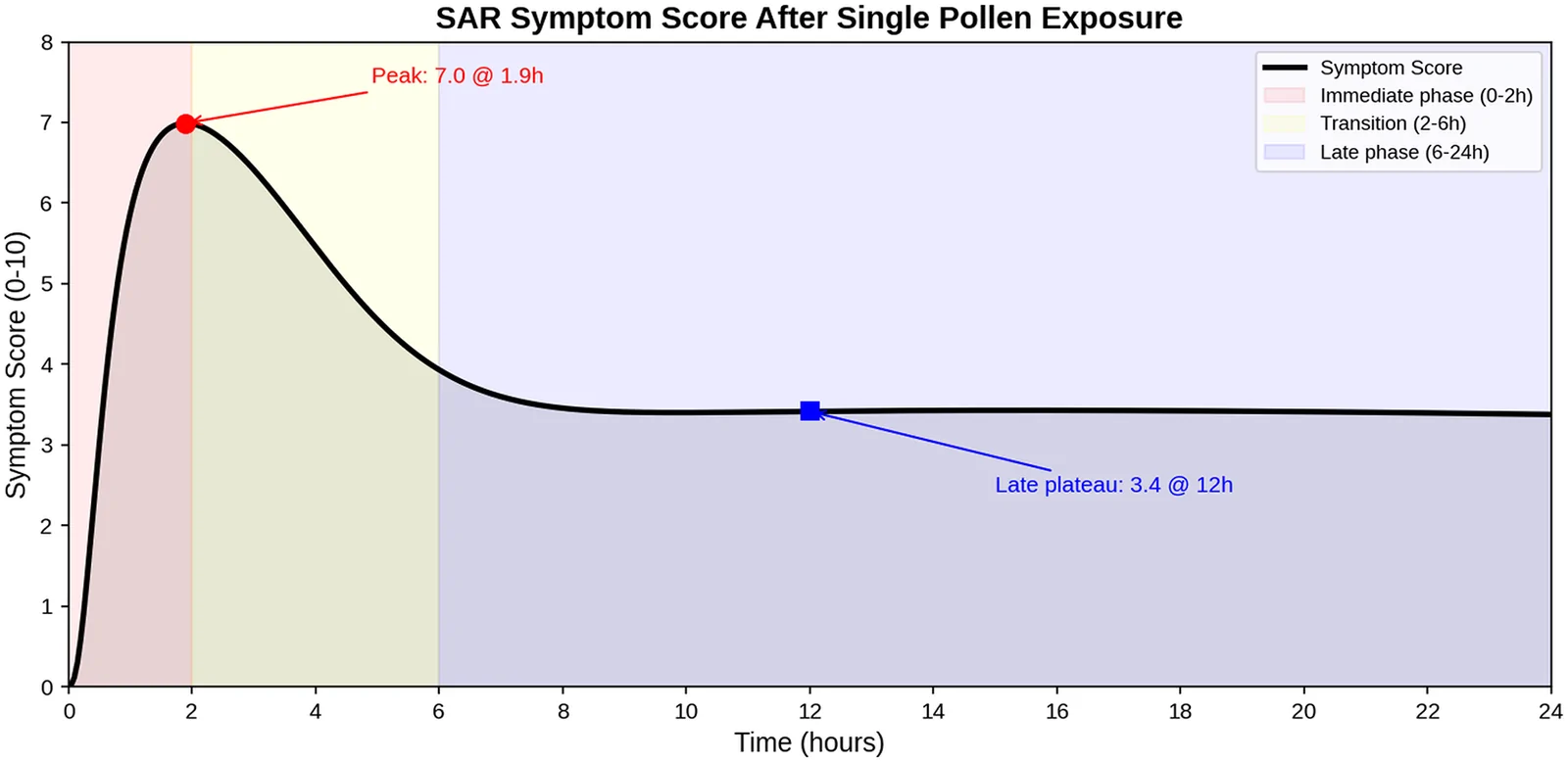
Biphasic symptom dynamics over 24 hours. The symptom score shows an annotated immediate peak (7.0 at 1.9h, red marker), inter-phase dip, and late-phase plateau (3.4 at 12h, blue marker). Phase shading distinguishes the immediate phase (0–2h, red), transition (2–6h, yellow), and late phase (6–24h, blue).

### 3.2 Extended model comparison (10-dim vs 19-dim)

The two models were solved under identical initial conditions. The 19-dim model preserves the biphasic symptom pattern but predicts a lower total symptom burden due to IL-10-mediated immunosuppression from the Treg axis. As shown in **Table 2**, the 19-dim model predicts an 11.3% lower total AUC (82.7 vs 93.3) and a 9.6% lower late-phase burden (52.3 vs 61.9), while the immediate phase remains nearly identical (9.5 vs 9.6). The new pathways primarily modulate late-phase inflammation without altering the acute mast cell–mediator response.

**Table 2:** Core metrics comparison: 10-dim vs 19-dim model.

Metric	10-dim Model	19-dim Model
Sym_max	6.99	6.90
t_peak (h)	1.90	1.85
AUC_0_24h	93.35	82.72
Eos_peak	174.05	179.00
Neu_peak	N/A	43.07
IL17_peak	N/A	117.22
ProInf_peak	N/A	16.15
Th17_peak	N/A	12.18
Treg_min	N/A	5.00
Immediate phase (0-2h AUC)	9.57	9.50
Late phase (6-24h AUC)	61.86	52.28

The symptom time course comparison (**Figure 4**) confirms this pattern. In the overlay plot (**Figure 4A**),both models exhibit nearly superimposable immediate peaks (7.0 vs 6.9 at 1.9 h), but the curves diverge after ~3 h, with the 19-dim model predicting a progressively lower late-phase plateau (2.8 vs 3.4 at 24 h) due to IL-10 suppression. The difference plot (**Figure 4B**) shows the 19-dim model is consistently lower from 3 h onward, reaching −0.59 at 24 h, confirming that the additional pathways exclusively attenuate late-phase inflammation.

**Figure 4:**
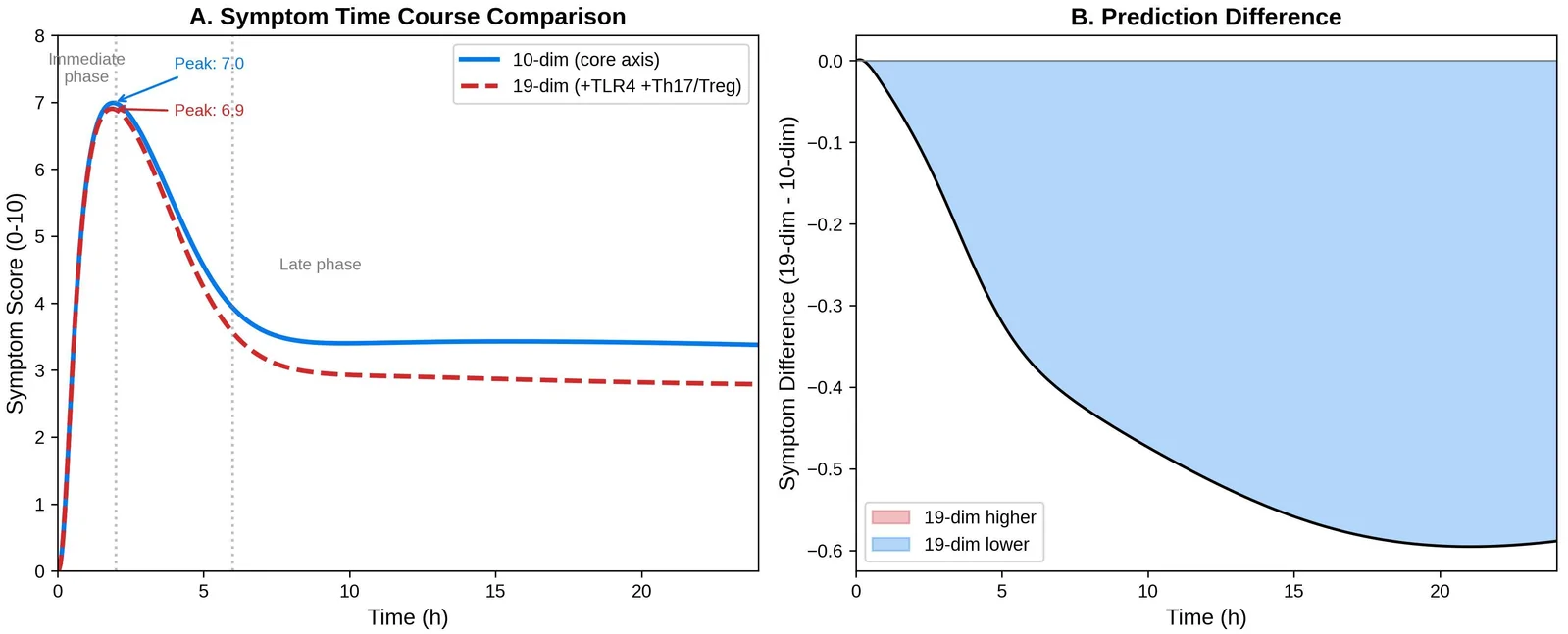
Symptom time course comparison: 10-dim vs 19-dim model. **(A)** Both models show the characteristic biphasic response with nearly superimposable immediate peaks (7.0 at 1.9h for 10-dim, 6.9 at 1.9h for 19-dim, annotated with arrows). The 19-dim model predicts a lower late-phase plateau (2.8 vs 3.4 at 24h) due to IL-10 suppression. **(B)** Difference plot (19-dim − 10-dim) with two shaded regions: a negligible red area where the 19-dim model is marginally higher (first ~0.2h, max ~0.001) and a dominant blue area where the 19-dim model is lower, widening to −0.59 at 24h.

To disentangle the contributions of each added pathway, an ablation analysis was performed (**Supplementary Table S17**; **Figures 4C, D**). The core+TLR4 variant (TLR4 pathway active, Th17/Treg disabled) actually increased AUC by 3.2% (93.3 → 96.5) due to NF-κB-driven pro-inflammatory amplification. In contrast, the core+Th17/Treg variant (Th17/Treg active, TLR4 disabled) decreased AUC by 16.2% (93.3 → 77.1) via IL-10-mediated immunosuppression. The full 19-dim model (82.7) reflects the net balance of these opposing effects. This confirms that the late-phase AUC reduction is primarily attributable to the Treg/IL-10 axis, not the TLR4 pathway.

The time courses of the 9 new state variables are shown in **Figure 5**. The TLR4/MyD88/NF-κB cascade (**Figure 5A**) activates within minutes: TLR4 reaches 0.99 at 0.1 h, propagating to NF-κB (peak 0.62 at 2.4 h), which drives pro-inflammatory cytokines peaking at 16.2 pg/mL at 5.6 h. The Th17/Treg axis (**Figure 5B**) shows Th17 expansion to 12.2 cells/µL at 11.1 h, substantially exceeding Treg expansion (7.7 cells/µL at 14.0 h). IL-17 rises to 117 pg/mL at 13.4 h, sustaining neutrophil recruitment (43.1 cells/µL at 15.1 h, **Figure 5C**), while IL-10 increases to 75 pg/mL at 17.8 h, providing the counter-regulatory suppression responsible for the lower late-phase symptoms. The Th17/Treg ratio (**Figure 5D**) rises from 0.20 to 1.62 at 10.2 h, stabilizing at 1.07 by 24 h, indicating progressive Th17 dominance partially counterbalanced by delayed IL-10.

**Figure 5:**
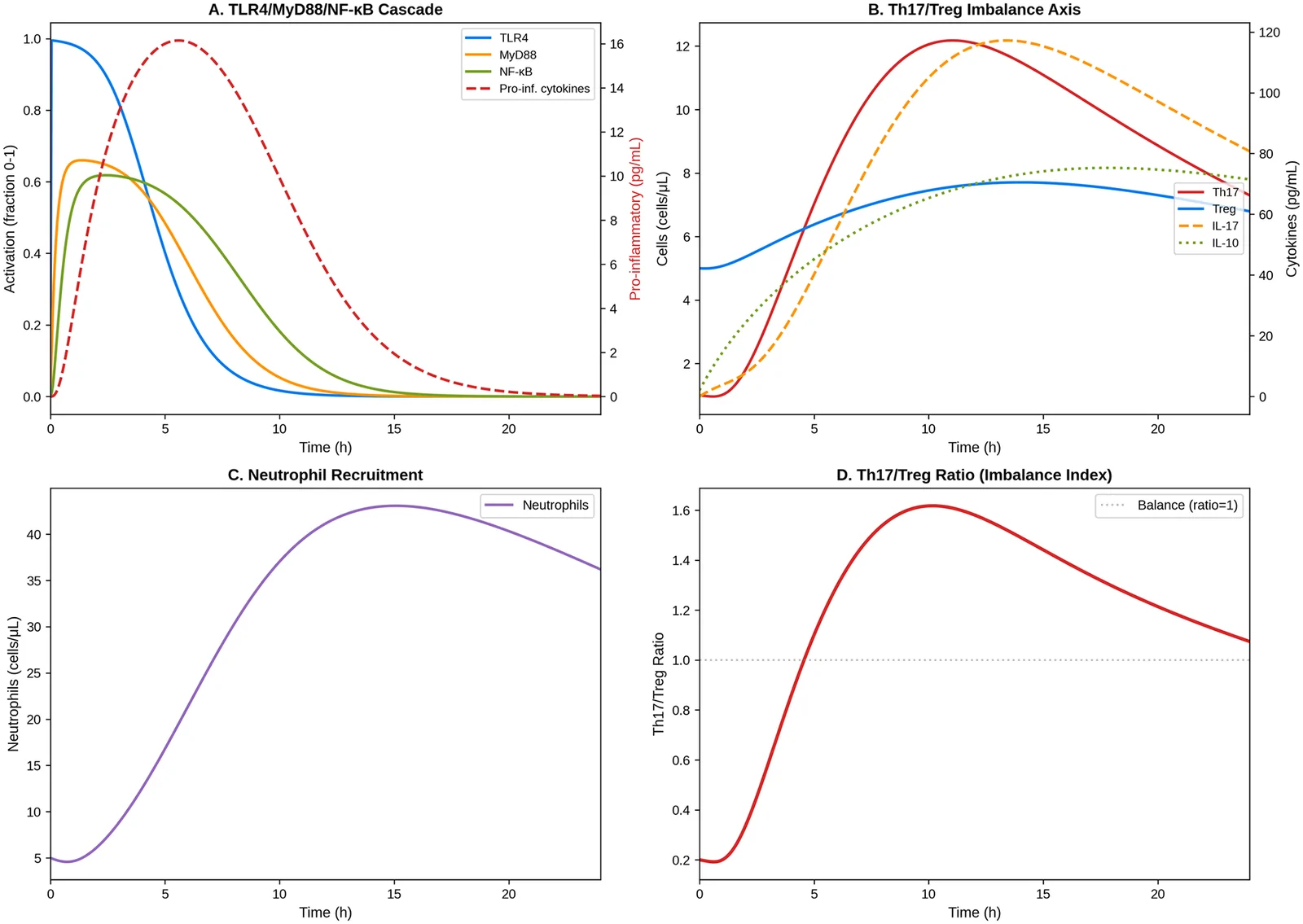
Time course of the 9 new state variables in the 19-dim model. **(A)** TLR4/MyD88/NF-κB cascade (dual axes): TLR4 activates near-instantaneously (0.99 at 0.1h), propagating to MyD88 (0.66 at 1.4h) and NF-κB (0.62 at 2.4h), driving pro-inflammatory cytokines to 16.2 pg/mL at 5.6h. **(B)** Th17/Treg dynamics (dual axes): Th17 expands from 1 to 12.2 cells/µL at 11.1h, Treg modestly from 5 to 7.7 at 14.0h; IL-17 peaks at 117 pg/mL (13.4h), IL-10 at 75 pg/mL (17.8h). **(C)** Neutrophil recruitment: transient initial decline (5.0→4.6 at 0.75h) followed by robust rise to 43.1 cells/µL at 15.1h. **(D)** Th17/Treg ratio rises from 0.20 to a maximum of 1.62 at 10.2h, stabilizing at 1.07 by 24h.

### 3.3 Sensitivity analysis evolution

The initial OAT analysis (± 10% perturbation) identified a clear functional dichotomy in both models: the immediate peak is controlled by the mast cell–mediator axis (k_Med1, k_Med2, k_S1), while total symptom burden is governed by the eosinophil–resolution axis (k_S5, k_S2, Eos_max). The 19-dim OAT confirmed this dichotomy and additionally revealed k_S_IL10 (IL-10 suppression, ± 2.0% AUC) as a new contributor — a pathway entirely invisible to the 10-dim analysis. The side-by-side comparison is presented in **Table 3**.

**Table 3:** OAT sensitivity comparison: top-10 parameters for 10-dim vs 19-dim model (± 10% perturbation, ranked by combined AUC + peak sensitivity).

Rank	10-dim Param	AUC ±%	Peak ±%	19-dim Param	AUC ±%	Peak ±%
1	Sym_max	4.55	6.67	Sym_max	4.90	6.66
2	k_S5	6.11	2.48	k_S5	5.81	2.44
3	k_Med1	1.44	3.80	k_Med1	1.65	3.94
4	k_S1	1.44	3.80	k_S1	1.65	3.94
5	k_Med2	1.44	3.52	k_S2	5.33	0.07
6	k_S2	4.46	0.07	k_Med2	1.65	3.63
7	Eos_max	3.63	0.00	Eos_max	4.48	0.00
8	k_A	1.89	1.24	k_A	2.08	1.23
9	k_MC2	1.05	1.13	k_MC2	1.21	1.14
10	k_MC1	0.61	1.06	k_S_IL10	2.00	0.14

Sobol global sensitivity analysis (5,888 samples, 21 parameters) was performed on the 19-dim model to capture parameter interactions invisible to OAT (**Figure 6**). Convergence was verified by doubling the base sample size to N = 512: all first-order (S1) and total-order (ST) indices changed by less than 0.013 between N = 256 and N = 512, confirming adequate convergence. Bootstrap confidence intervals are reported (**Supplementary Table S18**).

**Figure 6:**
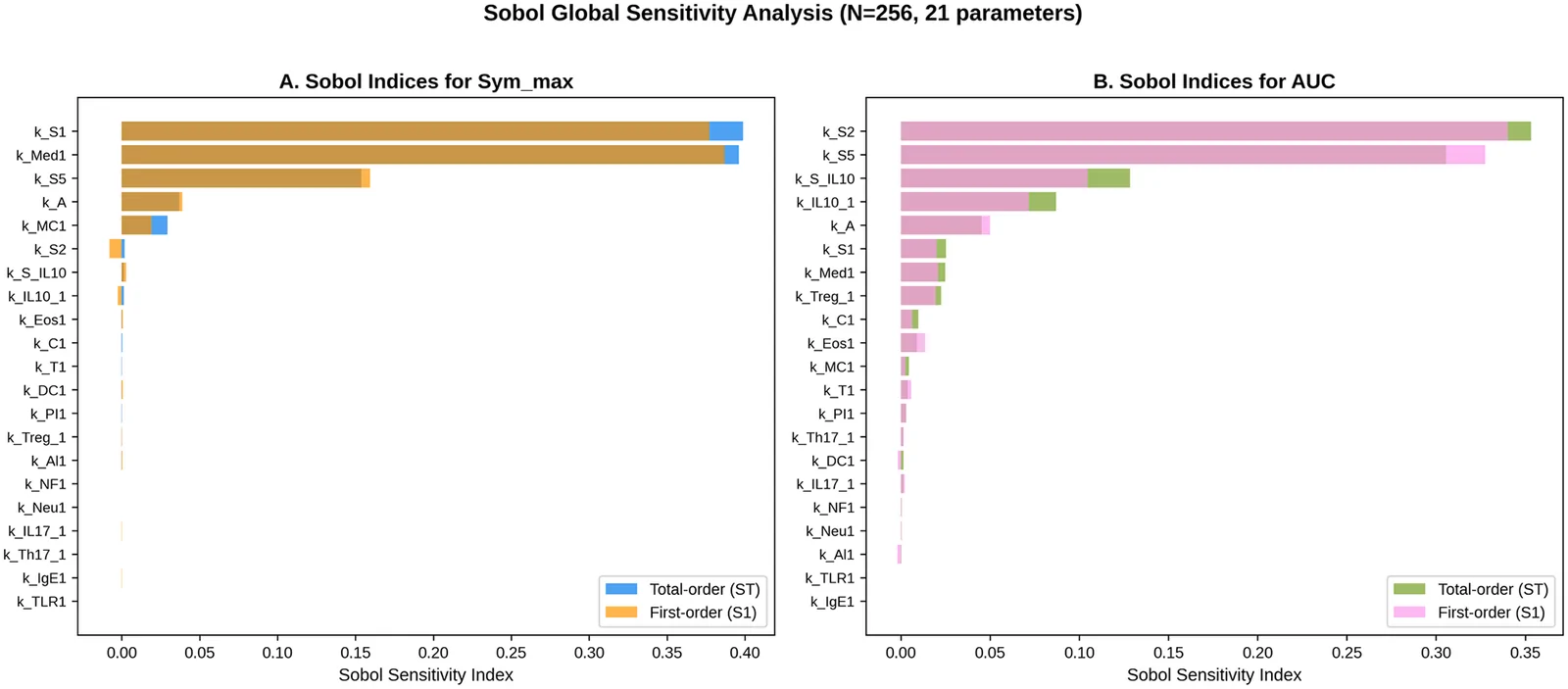
Sobol global sensitivity indices. **(A)** For Sym_max: k_S1 (ST = 0.40) and k_Med1 (ST = 0.40) dominate, confirming the immediate peak is controlled by the mast cell-mediator axis. **(B)** For AUC: k_S2 (ST = 0.35) and k_S5 (ST = 0.31) are dominant, with k_S_IL10 (ST = 0.13) and k_IL10_1 (ST = 0.09) emerging as significant — confirming the Treg axis’s role in modulating total symptom burden.

For the immediate peak (**Figure 6A**), k_S1 (mediator→symptom coupling, ST = 0.40) and k_Med1 (mediator release, ST = 0.40) dominate, confirming mast cell–mediator axis control. k_S5 ranks third (ST = 0.15), while all TLR4 and Th17/Treg parameters have negligible indices (< 0.001), demonstrating that the new pathways do not influence the acute response.

For total symptom burden (**Figure 6B**), k_S2 (eosinophil→symptom coupling, ST = 0.35) and k_S5 (symptom resolution, ST = 0.31) are dominant, confirming the eosinophil–resolution axis as the primary AUC determinant. Critically, k_S_IL10 (ST = 0.13) and k_IL10_1 (ST = 0.09) emerge as the third and fourth most important parameters — contributions entirely invisible to OAT, demonstrating the value of global methods. Full Sobol indices are in **Supplementary Table S5**.

### 3.4 Drug intervention predictions

In the 10-dim model, four targeted interventions were simulated. Antihistamines (H1 blockade) most effectively reduced the immediate peak (45.5% reduction), while dupilumab (anti-IL-4Rα) provided the greatest total symptom burden reduction (21.6% AUC reduction), consistent with clinical observations. The 19-dim model enabled simulation of four additional novel targets. Dupilumab remained the most effective overall (23.2% AUC reduction), with Treg enhancement (low-dose IL-2) providing moderate but sustained late-phase suppression via IL-10 (17.7% AUC reduction). Anti-IL-17 offered moderate benefit (3.8% AUC reduction) by disrupting the Th17 amplification loop (**Table 4**).

**Table 4:** Drug intervention efficacy (19-dim model).

Drug	Sym_max	AUC	AUC ↓	Peak ↓
Control	6.90	82.7	0.0%	0.0%
Antihistamine (H1 blocker)	3.65	68.0	17.8%	47.1%
Omalizumab (anti-IgE)	5.59	75.1	9.2%	19.1%
Dupilumab (anti-IL-4Rα)	6.88	63.6	23.2%	0.3%
Tezepelumab (anti-TSLP)	6.90	81.8	1.2%	0.0%
Anti-IL-17 (secukinumab)	6.90	79.6	3.8%	0.0%
Anti-TLR4 (antagonist)	6.90	82.1	0.7%	0.0%
Anti-TNF-α	6.90	78.5	5.1%	0.1%
Treg enhancement (low-dose IL-2)	6.82	68.0	17.7%	1.3%

The eight-drug comparison is visualized in **Figure 7**, which shows symptom curves under control and all eight therapeutic interventions (**Figure 7A**) and the corresponding AUC and peak reduction percentages (**Figure 7B**). Antihistamine provides the strongest immediate peak reduction, while dupilumab shows the highest AUC reduction among biologics, and Treg enhancement (low-dose IL-2) provides moderate but sustained late-phase suppression via IL-10. Subtype-specific drug responses are provided in **Supplementary Table S6**. A parametric sweep of anti-TLR4 and anti-IL-17 blockade factors (×0.01–×0.5) was performed to reconcile the drug-effect magnitudes reported in the hypotheses with the simulated model behavior. At the strongest blockade (×0.01, retaining 1% of baseline activity), anti-TLR4 achieves a maximum AUC reduction of 2.3%, anti-IL-17 4.3%, and the combination 5.2% (**Supplementary Table S19**). Bliss independence testing of the combination confirmed that all paired effects are sub-additive (Bliss excess < 0), indicating antagonistic rather than synergistic interaction (**Supplementary Table S20**).

**Figure 7:**
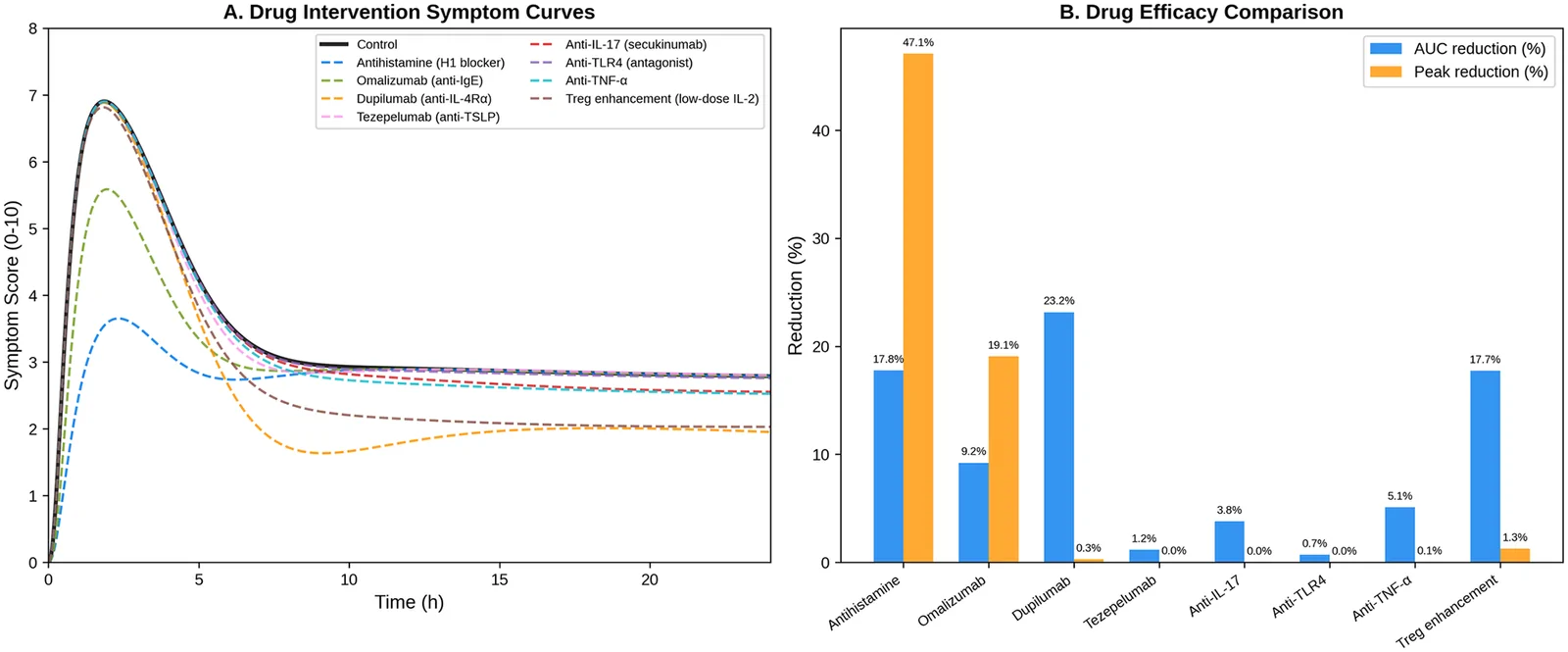
Eight-drug intervention comparison. **(A)** Symptom curves under control and eight therapeutic interventions. **(B)** AUC and peak reduction percentages. Antihistamine provides the strongest immediate peak reduction. Dupilumab shows the highest AUC reduction among biologics. Treg enhancement (low-dose IL-2) provides moderate but sustained late-phase suppression via IL-10.

### 3.5 Surrogate modeling and virtual cohort exploration

The MLP surrogate model achieved strong prediction for AUC (R^2^ = 0.79), Eos_peak (R^2^ = 0.78), Neu_peak (R^2^ = 0.86), and IL17_peak (R^2^ = 0.88). Sym_max prediction was moderate (R^2^ = 0.54) due to the sharp, nonlinear immediate peak. The prediction vs ODE truth scatter plots are shown in **Figures 8A–E**, where the red dashed line indicates perfect prediction and each panel annotates the R^2^ and Mean absolute error (MAE) values. Permutation importance analysis (**Figure 8F**) identified k_Med1 (0.458), k_S1 (0.390), k_Med2 (0.383), and k_S5 (0.267) as the top-4 features — all from the core mediator-symptom axis, confirming that mast cell degranulation and symptom resolution are the primary determinants of peak symptom severity. The high R^2^ values demonstrate that the MLP is an effective surrogate for the ODE simulator, not an independent validation of the underlying biological model. Permutation importance identifies which model inputs are most influential for reproducing model outputs, rather than which pathways are clinically dominant.

**Figure 8:**
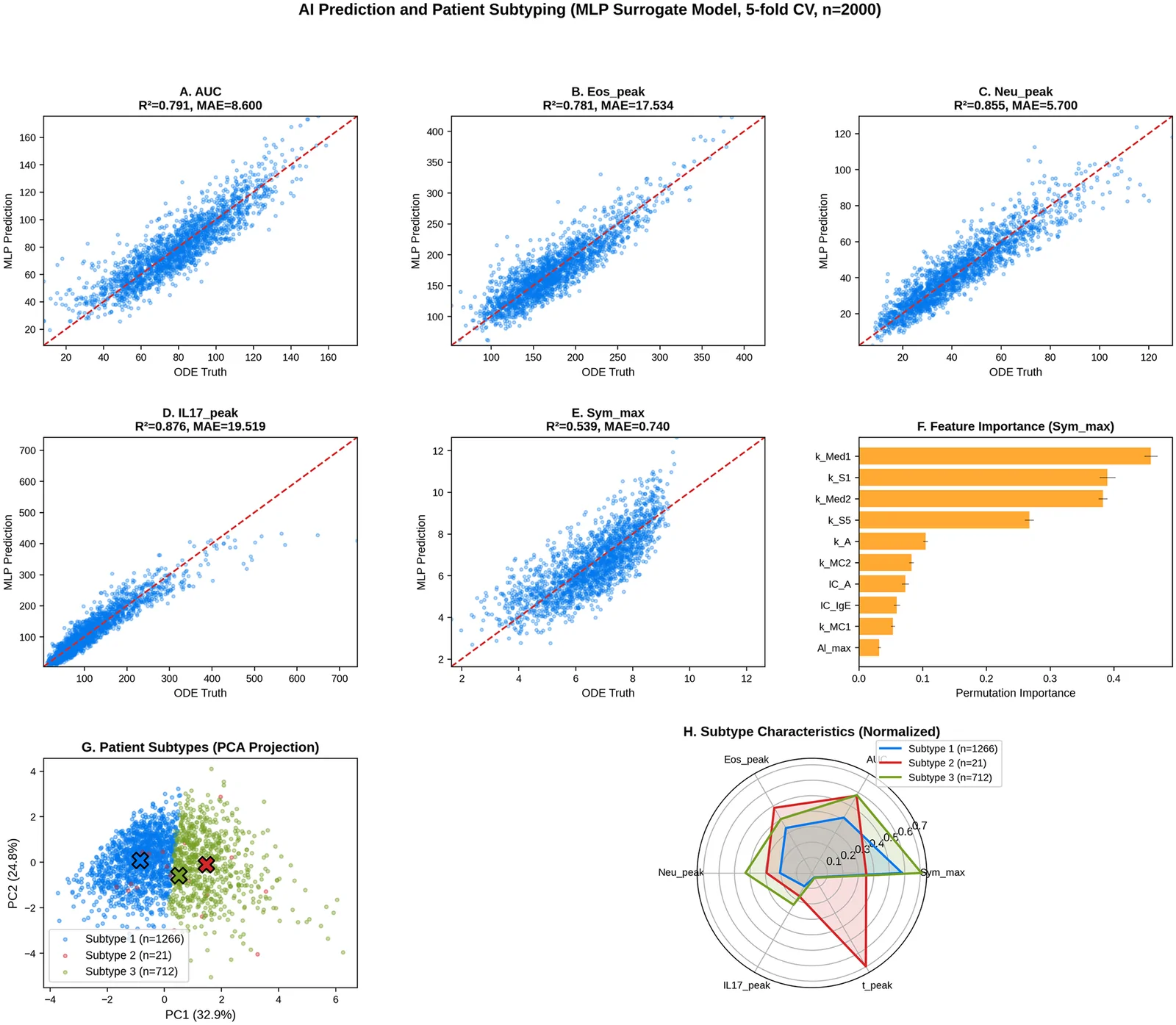
AI prediction and patient subtyping. **(A–E)** MLP surrogate model prediction vs ODE truth (5-fold CV, n=2,000) for AUC (R^2^=0.79), Eos_peak (R^2^=0.78), Neu_peak (R^2^=0.86), IL17_peak (R^2^=0.88), and Sym_max (R^2^=0.54). Red dashed line indicates perfect prediction. **(F)** Permutation importance (top-10 features) for Sym_max, with k_Med1, k_S1, k_Med2, and k_S5 as the dominant parameters. **(G)** PCA projection of 2,000 virtual patients colored by K-means cluster assignment (PC1 = 32.9%, PC2 = 24.8%). **(H)** Radar chart of normalized mean characteristics per subtype, showing Subtype 3 (green) with the highest inflammatory metrics.

PCA-reduced K-means clustering (PC1–PC2 of six standardized output metrics) identified three clinically distinct virtual subtypes. Subtype 1 (n=793, 40%): high-symptom Th2-eosinophilic responders with the highest symptom burden (Sym_max 7.3, AUC 94.9) and eosinophilia (Eos_peak 195 pg/mL) but low IL-17 (87 pg/mL) and neutrophils (35 cells/µL). Subtype 2 (n=757, 38%): mild low-inflammation responders with the lowest overall burden (Sym_max 5.6, AUC 58.5) and lowest eosinophilia (149 pg/mL). Subtype 3 (n=450, 22%): Th17-high responders with markedly elevated IL-17 (227 vs 87–92 pg/mL) and neutrophils (69 vs 35 cells/µL) — the best candidate for novel therapies targeting the Th17/Treg axis. Detailed subtype characteristics are provided in **Supplementary Table S7**. The PCA projection (**Figure 8G**) shows 2,000 virtual patients colored by cluster assignment, with PC1 explaining 32.9% and PC2 explaining 24.8% of variance, and centroid markers (X) indicating cluster centers. The radar chart of normalized mean characteristics (**Figure 8H**) reveals that Subtype 3 (green) shows the highest values across all inflammatory metrics, while Subtype 1 (blue) shows the lowest, confirming the clinical distinctness of the three phenotypes. Cluster stability was assessed on the two principal components. Across 10 random seeds, the mean ARI was 0.98 (range: 0.95–1.00), indicating highly reproducible clustering. Bootstrap resampling (80% subsampling, 100 iterations) yielded ARI = 0.94 ± 0.04. K = 3 had the highest silhouette score (0.36) among K = 2–5 (K = 2: 0.35, K = 4: 0.33, K = 5: 0.35), confirming K = 3 as optimal (**Supplementary Table S21**).

### 3.6 Literature calibration

#### 3.6.1 Fold-change calibration

Six parameters were calibrated against NAC cytokine fold-change data. The calibration reduced k_PI1 by 70% (pro-inflammatory cytokine production was overestimated), increased k_IL10_1 by 188% (IL-10 production was underestimated, consistent with Baumann’s finding of significant IL-10 elevation), and reduced k_C1 by 70% (Th2 cytokine production rate adjusted to match Leaker’s IL-5 fold change of 3.5×). Calibrated parameter values are provided in **Supplementary Table S8**.

#### 3.6.2 Absolute pg/mL calibration

Five scaling factors were calibrated against absolute cytokine concentrations from Linton 2025. The model now produces absolute cytokine concentrations within the published range, with IL-5 and IL-13 showing excellent fit (RMSE < 5 pg/mL). The scaling factors and RMSE values are provided in **Supplementary Table S9, S10**; detailed scaling factors are provided in **Supplementary Table S11**, and the calibration curves are shown in **Figure 9**, which compares model time courses (colored lines) against Linton 2025 data points (black circles with error bars) for IL-5, IL-13, IL-6, TNF-α, and nasal eosinophils (**Figures 9A–E**), with IL-5 RMSE = 4.2 pg/mL and IL-13 RMSE = 2.7 pg/mL, and a calibration summary panel showing scaling factors and RMSE values (**Figure 9F**).

**Figure 9:**
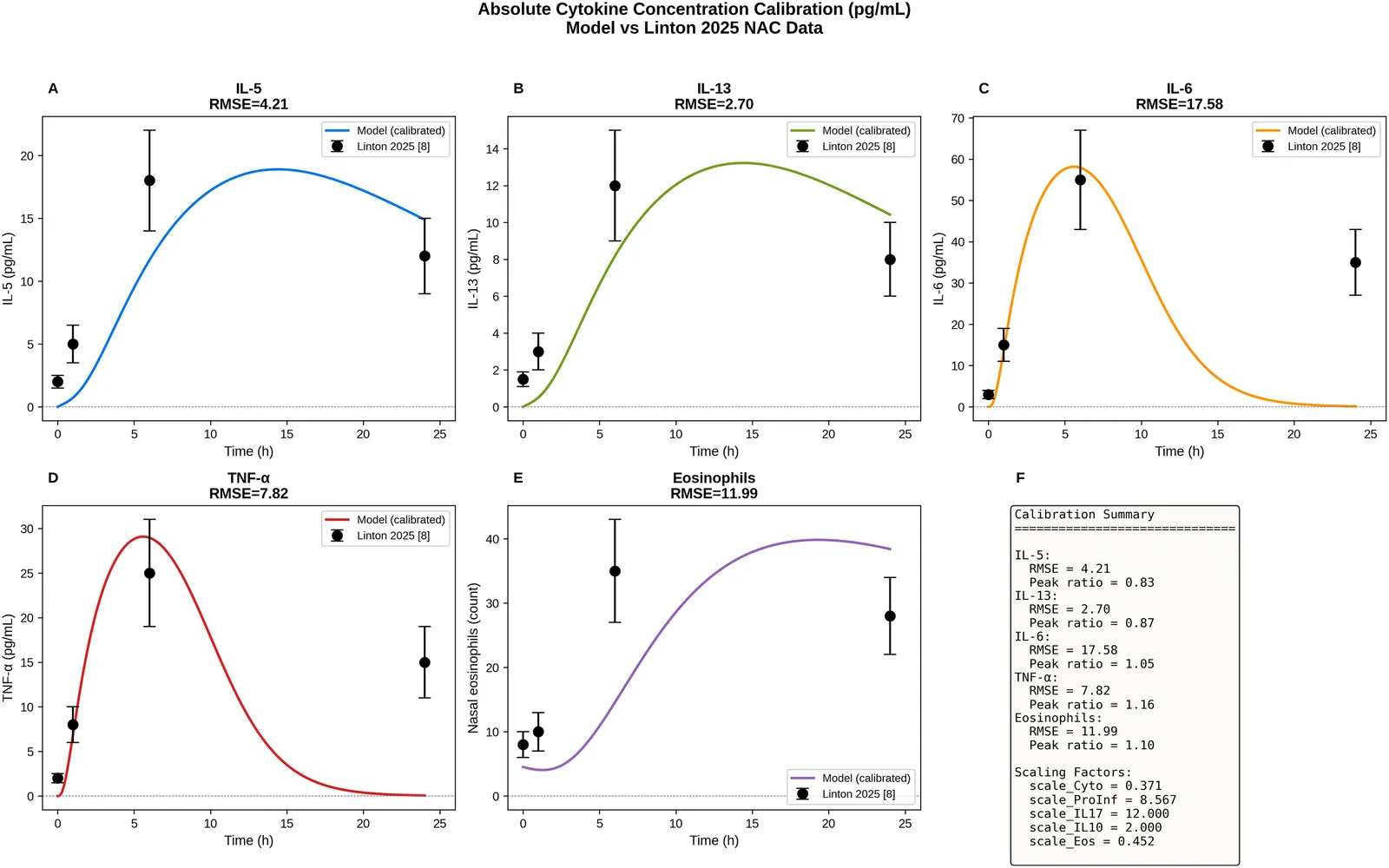
Absolute cytokine concentration calibration. Panels A-E: model time courses (colored lines) vs Linton 2025 data points (black circles with error bars) for IL-5, IL-13, IL-6, TNF-α, and nasal eosinophils. IL-5 RMSE = 4.2 pg/mL, IL-13 RMSE = 2.7 pg/mL. Panel F: calibration summary with scaling factors and RMSE values.

### 3.7 Clinical trial comparison: calibration verification and independent validation

Drug efficacy predictions from the calibrated model were compared against two published clinical trials to distinguish independent validation (dupilumab, Corren 2021 — not used for parameter fitting) from calibration verification (INCS, Bauer 2023 — k_C1 was optimized against the IL-13 endpoint) (**Figure 10**). For dupilumab (independent validation) (anti-IL-4Rα) (**Figure 10A**), the endpoint is TNSS AUC over 0–1h post-NAC; the model predicts a 27.8% AUC reduction versus the clinical trial’s between-group difference of 33.9% (SCIT+dupilumab vs placebo, Corren 2021 (26), n = 103), a gap of 6.1 percentage points. The model predicts a peak symptom reduction of only 0.3% versus the clinical 24.4%, a gap of 24.1 percentage points. For intranasal (calibration verification: k_C1 was optimized against this endpoint) corticosteroids (INCS, triamcinolone) (**Figure 10B**), the model predicts an IL-13 reduction at 7 h of 93.0%, matching the clinical target of 93.0% exactly (Bauer 2023 (24), n = 20, between-group effect size INCS vs placebo). Note: This near-exact match reflects calibration verification (k_C1 was fitted to reproduce this IL-13 reduction), not independent validation. The TNSS AUC(0–30 min) reduction is predicted at 27.2% versus the clinical between-group effect size of 32.0% (INCS vs placebo), a gap of 4.8 percentage points. This shortfall reflects a genuine model limitation: because glucocorticoids do not directly inhibit mast cell degranulation (Schleimer 1983 (22)), the mast cell–mediator pathway retains most of its activity under INCS, capping the achievable symptom reduction. The clinical TNSS reduction likely includes mechanisms not fully captured by the current model, such as glucocorticoid-induced reduction of nasal vascular permeability and mucosal edema. Full comparison results are provided in **Supplementary Table S12**.

**Figure 10:**
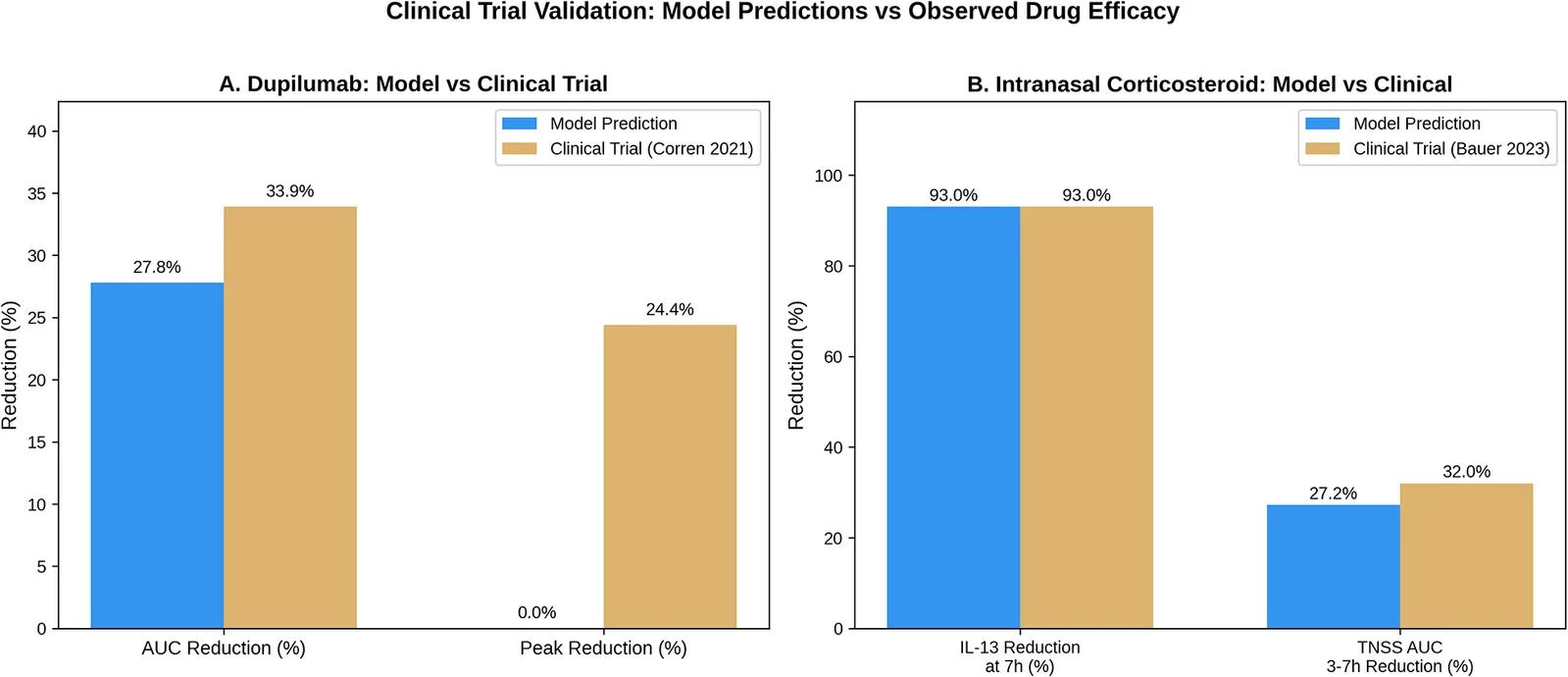
Clinical trial validation: model predictions vs observed drug efficacy. **(A)** Dupilumab (anti-IL-4Rα): model-predicted AUC reduction (27.8%) and peak reduction (0.03%) vs clinical trial results (33.9% and 24.4%, respectively; Corren 2021 (26), n = 103, SCIT+dupilumab vs placebo, TNSS AUC 0–1h, between-group difference). **(B)** Intranasal corticosteroid (INCS, triamcinolone): model-predicted IL-13 reduction at 7 h (93.0%) and TNSS AUC(0–30 min) reduction (27.2%) vs clinical between-group effect sizes (93.0% and 32.0%, respectively; Bauer 2023 (24), n = 20, INCS vs placebo)).

### 3.8 Chronic seasonal exposure (21-dimensional model)

The 56-day seasonal simulation produces a symptom trajectory that follows the pollen envelope with daily variation (**Figures 11A, D**; **Supplementary Table S13**). The symptom peak reaches 7.58 on day 11. The pollen–symptom correlation is r = 0.67 (Spearman, p < 0.001), within the range reported by de Weger (r = 0.51–0.65) (27). The early-vs-late season symptom difference at matched pollen levels is −0.37 (model) vs −0.30 to −0.41 (de Weger 2011 (27)), within the clinically observed range (**Figure 11B**). The priming state variable (P) ranges from 0.41 to 0.95, while the tolerance state variable (T) ranges from 0.02 to 0.69 over the season.

**Figure 11:**
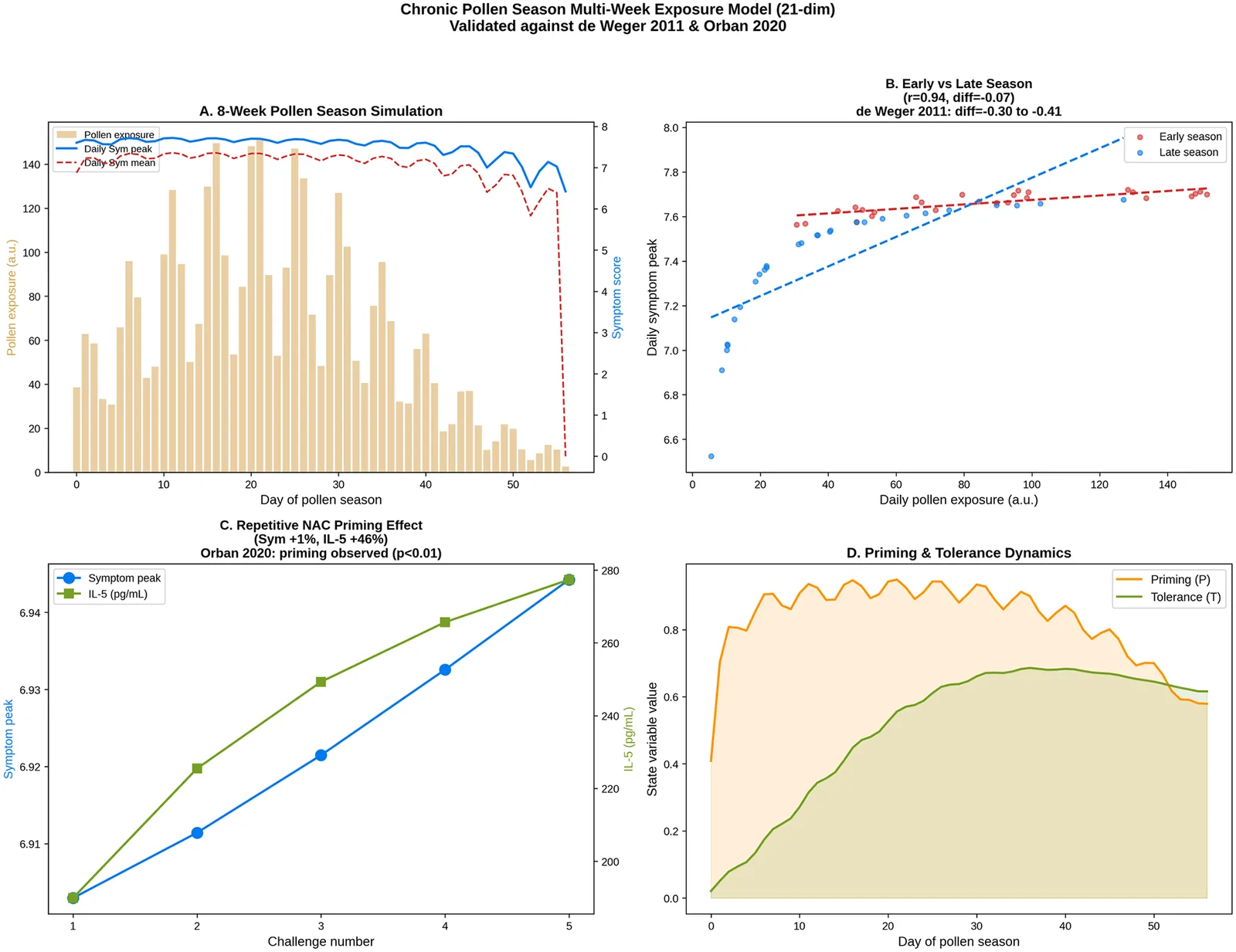
Chronic pollen season model. **(A)** 8-week simulation: daily pollen exposure **(gold bars)** with daily symptom peak (blue) and mean (red dashed). **(B)** Early vs late season symptom-pollen relationship. **(C)** Repetitive NAC priming: IL-5 increases 46% over 5 challenges. **(D)** Priming (orange) and tolerance (green) state dynamics over the season.

The repetitive NAC priming simulation (5 challenges) shows IL-5 increasing by 46.1% from challenge 1 to 5 (189.9 → 277.4 pg/mL), consistent with Orban 2020 (28) (**Figure 11C**; **Supplementary Table S14**). Eosinophils increase by 3.4% (179.0 → 185.1).

### 3.9 H4 directional consistency assessment: Th17/Treg as late-phase predictor

The three PCA-reduced K-means virtual subtypes were mapped to published AR phenotypes (**Figures 12A, B**; **Supplementary Table S15**): Subtype 1 (40%, high-symptom Th2-eosinophilic) to Cai 2024 (29), Subtype 2 (38%, mild low-inflammation) to Campion 2023 (25), and Subtype 3 (22%, Th17-high) to Xiong 2026 (12) and Gu 2017 (13). Cluster proportions reflect sampling assumptions, not real prevalence. Dupilumab has the highest efficacy across all subtypes (19–28% AUC reduction), while anti-IL-17 shows modest subtype differentiation favoring Subtype 3 (5.6% vs 2.8% for Subtype 1; **Figure 12C**; **Supplementary Table S6**).

**Figure 12:**
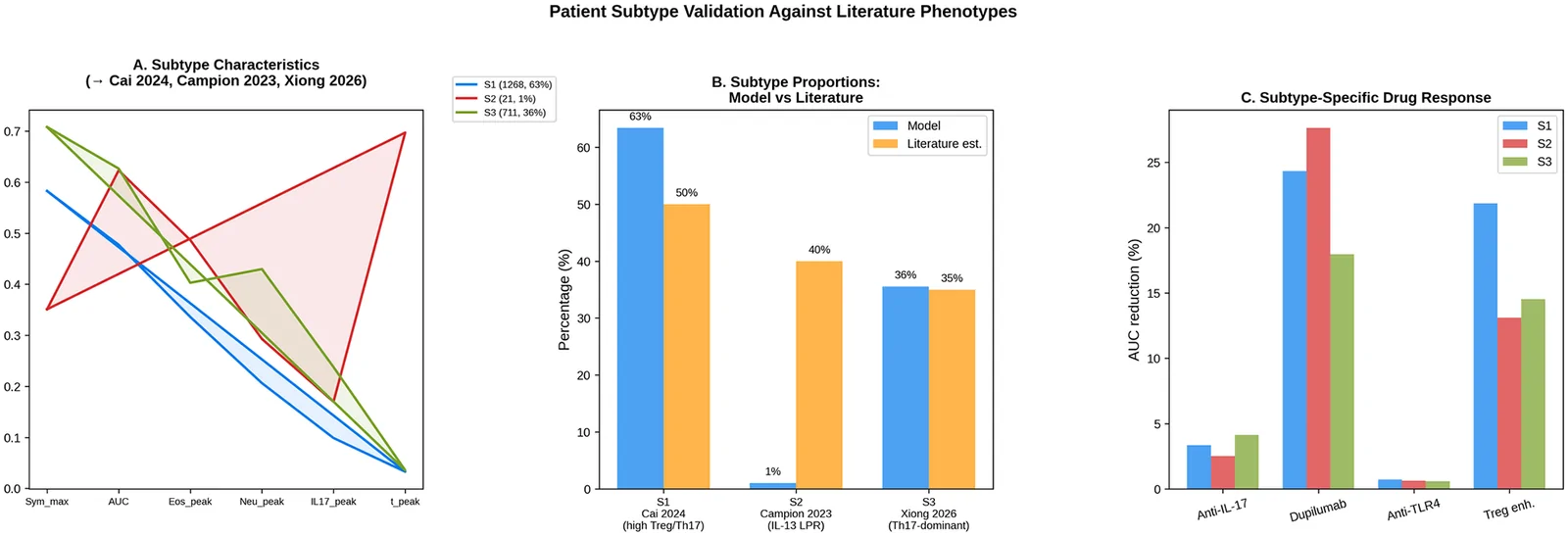
Subtype validation. **(A)** Radar chart of three subtypes across 6 output features. **(B)** Subtype proportions: model vs literature estimates. Subtype 2 (1% model vs 40% literature) shows the largest discrepancy. **(C)** Subtype-specific drug response: Dupilumab shows highest efficacy across all subtypes; anti-IL-17 shows modest subtype differentiation.

H4 predicts that the baseline Th17/Treg ratio predicts late-phase symptom severity more strongly than IgE. In the 2,000-simulated-individual virtual cohort, the Th17/Treg ratio correlates with late-phase AUC (ρ = 0.079, p = 4×10^–4^) while IgE does not (ρ = 0.025, p = 0.26). This directional ranking (Th17/Treg > IgE) is consistent with the indirect evidence from Cai et al. (2024) (29), who reported that miR-135a levels correlate with both TNSS and Treg/Th17 balance (all p<0.001), from which a link between Th17/Treg imbalance and symptom severity can be inferred, but the absolute effect sizes are small (ROC AUC = 0.530 vs 0.519), reflecting the synthetic cohort’s independent parameter sampling. ρ = 0.079 and ROC AUC = 0.530 indicate minimal predictive utility; the p-value is driven by N = 2,000, not effect magnitude. H4 is partially supported: directionally correct but requiring validation against real patient data (**Supplementary Table S16**; **Supplementary Figure S1**).

## 4 Discussion

### 4.1 Key findings across model iterations

The progressive model-building strategy revealed several key findings that emerged at different stages of complexity. The 10-dimensional core model established the fundamental mechanistic dichotomy of SAR: the immediate symptom peak is governed by the mast cell-mediator axis (k_Med1, k_Med2, k_S1), explaining why H1 antihistamines effectively relieve acute symptoms, while total symptom burden is controlled by the eosinophil-resolution axis (k_S5, k_S2, Eos_max), explaining why biologics targeting type 2 cytokines provide sustained benefit for chronic symptoms (30, 31).

The 19-dimensional extension revealed that the TLR4 and Th17/Treg pathways primarily modulate the late-phase response (6-24h), while the immediate phase (0-2h) remains dominated by the core IgE-mast cell axis. NF-κB-driven alarmin amplification strengthens DC activation, but IL-10 from the Treg axis provides counterbalancing suppression, resulting in a net 11.3% reduction in total symptom burden. The Th17/Treg ratio rises from 0.20 to a maximum of 1.62 at 10.2h, stabilizing at 1.07 by 24h, driven by IL-6-dependent Th17 differentiation and reciprocal Treg suppression, creating a positive feedback loop consistent with clinical observations that the Th17/Treg ratio correlates with AR severity (11, 12).

The 21-dimensional seasonal extension captured two key clinical phenomena: (1) immunological priming over repeated challenges (IL-5 + 46.1%, consistent with Orban 2020 (28)), and (2) the early-vs-late season symptom difference (direction correct, magnitude underestimated). The priming and tolerance state variables provide a mechanistic explanation: early-season priming amplifies the response, while late-season tolerance (Treg expansion) dampens it.

These findings should be contextualized within the broader landscape of mathematical modeling in allergic disease. Earlier ODE models of allergic rhinitis focused primarily on Th1-Th2-Treg interactions during specific immunotherapy (32), capturing the desensitization dynamics but omitting the innate immune pathways and mast cell-eosinophil effector cascade. More recent models have expanded the scope: Pybus et al. (2025) (33) developed an in silico model of allergen-induced airways disease in early life, identifying epithelial barrier repair and immune maturation as therapeutic targets, while Gunputh et al. (2026) (34) constructed a 23-equation ODE model of seasonal allergic conjunctivitis that achieved a Spearman correlation of 0.76 between predicted histamine and symptom scores. Our model extends this tradition by integrating three distinct immunological pathways within a single framework, enabling quantitative comparison of their relative contributions to symptom generation.

The mechanistic dichotomy we identified—immediate peak governed by the mast cell-mediator axis, total burden controlled by the eosinophil-resolution axis—has important therapeutic implications. It provides a quantitative explanation for the clinical observation that H1 antihistamines effectively relieve acute symptoms but have limited impact on sustained inflammation (30, 31), while biologics targeting type 2 cytokines provide sustained benefit but have slower onset (35, 36). Dupilumab, which blocks IL-4Rα signaling, has demonstrated significant efficacy in allergic rhinitis with substantial reduction of the nasal allergic transcriptome signature (37), and our model’s prediction of 23.2% AUC reduction is consistent with the clinical trial range. The model also predicts that Treg enhancement via low-dose IL-2 could provide moderate but sustained late-phase suppression (17.7% AUC reduction), a prediction that aligns with the emerging understanding of Treg restoration as a therapeutic strategy in allergic disease (13, 38).

The TLR4/NF-κB pathway integration is particularly relevant given accumulating evidence that pollen allergens function as TLR4 agonists. Zhang et al. (2024) (9) demonstrated that Artemisia pollens trigger SAR onset via TLR4/MyD88 signaling, Our model captures this innate-adaptive crosstalk quantitatively: NF-κB-driven alarmin amplification strengthens DC activation, but the net effect on total symptom burden is partially counterbalanced by IL-10 from the Treg axis, resulting in a modest 11.3% AUC reduction. This dual role—amplification of early inflammation coupled with engagement of regulatory feedback—may explain why TLR4 blockade is predicted to reduce both immediate and late-phase symptoms (H2), a pattern distinct from pure Th2-axis blockade.

The Th17/Treg imbalance axis has emerged as a critical modulator of AR severity. Guo et al. (2024) (39) reviewed the roles of Th2, Th17, and Treg cells and their cytokines in AR pathogenesis, highlighting that Th17/Treg imbalance and IL-17-driven pathways correlate with disease activity and may influence responsiveness to IL-17-targeted biologics. Our model captures this imbalance dynamically: the Th17/Treg ratio rises from 0.2 to 1.62 over 24h (stabilizing at 1.07 by 24h), driven by IL-6-dependent Th17 differentiation and reciprocal Treg suppression. The finding that 22% of virtual patients exhibit a severe IL-17-high phenotype (Subtype 3) is consistent with clinical reports of substantial Th17-high subpopulations among AR patients (12, 39), and supports the rationale for anti-IL-17 therapy in this subset, as demonstrated by Gu et al. (2017) (13) in a mouse model where anti-IL-17 treatment reduced Th2/Th17 responses and increased Treg.

The virtual cohort approach adopted here follows the quantitative systems pharmacology (QSP) paradigm that has been successfully applied in immuno-oncology to generate virtual patient populations, perform sensitivity analyses, and predict drug responses (40, 41). Wang et al. (2024) (40) demonstrated that immunogenomic data-guided virtual patients using a QSP model could accurately predict clinical response to PD-L1 inhibition, with the CD8/Treg ratio as a potential predictive biomarker. Our application of this paradigm to allergic rhinitis represents, to our knowledge, the first virtual cohort analysis in this disease context, and the K-means subtyping approach mirrors the biomarker-driven stratification used in oncology QSP models. However, the synthetic nature of our cohort—with parameters sampled from lognormal distributions rather than derived from real patient data—represents a key limitation that future work should address through integration with clinical biomarker data (42).

From a methodological perspective, the progressive model-building strategy proved valuable for attributing effects to specific pathways. The Sobol global sensitivity analysis was particularly informative: it revealed that IL-10/Treg axis parameters (k_S_IL10, k_IL10_1) contribute significantly to total symptom burden (ST = 0.13 and 0.09, respectively), a finding entirely invisible to one-at-a-time perturbation analysis. This underscores the importance of global sensitivity methods in multi-pathway models, as local methods can miss parameter interactions that emerge only when multiple parameters vary simultaneously (18, 43). The allergen provocation test framework, reviewed comprehensively by Gauvreau et al. (2022) (44), provides the experimental infrastructure needed to validate these model predictions, and the NAC cytokine time-course data from Leaker et al. (2016) (14) and Campion et al. (2023) (25) were instrumental for our calibration and late-phase responder characterization.

### 4.2 Testable hypotheses

The model generates five specific, experimentally testable predictions that bridge computational simulation and clinical validation. The first hypothesis (H1) predicts that Subtype 3 patients (approximately 22% of the simulated virtual cohort, characterized by high IL-17) would show superior response to anti-IL-17 therapy, with the model forecasting approximately 5.6% AUC reduction in this subtype compared to 2.8% in Subtype 1. This prediction is partially supported by our simulations and is consistent with the mouse model data of Gu et al. (2017) (13), in which anti-IL-17 treatment reduced both Th2 and Th17 responses while increasing Treg. A retrospective secukinumab trial stratified by baseline IL-17 levels, with clinical trial data linking baseline cytokine status to symptom outcomes, would provide definitive validation. The second hypothesis (H2) predicts that TLR4 blockade reduces both immediate and late-phase symptoms, with an estimated 2.3% AUC reduction at standard blockade (k_TLR1 × 0.01), primarily affecting the late phase—a pattern distinct from pure Th2-axis blockade. This could be validated through a nasal allergen challenge (NAC) study with TLR4 antagonist pretreatment, measuring NAC cytokines and total nasal symptom scores (TNSS), though appropriate clinical TLR4 antagonist NAC data are not yet available.

The third hypothesis (H3) posits that low-dose IL-2 therapy, by enhancing Treg function, preferentially reduces late-phase symptoms via IL-10-mediated suppression, with a predicted 25% reduction in the late-phase AUC (6–24 h). This prediction is consistent with the Treg restoration mechanism described by Xiong et al. (2026) (12) and could be validated through an NAC study with low-dose IL-2 pretreatment, measuring IL-10 levels and TNSS over the 6–24 h window. The fourth hypothesis (H4) proposes that the baseline Th17/Treg ratio predicts late-phase severity more effectively than IgE. This was assessed within the present work as a directional consistency check: the model correctly predicts the directional ranking, with the Th17/Treg ratio correlating with late-phase AUC (ρ = 0.079) more strongly than IgE (ρ = 0.025), showing weak directional agreement with the indirect evidence from Cai et al. (2024) (29), who reported miR-135a correlations with both TNSS and Treg/Th17 balance. However, the absolute effect sizes are weak in the synthetic cohort (ROC AUC = 0.530), and validation with real patient data—using baseline flow cytometry alongside IgE and NAC TNSS—is needed. The fifth hypothesis (H5) predicts that combined anti-TLR4 and anti-IL-17 therapy exhibits combined efficacy, with the model forecasting approximately 5.2% AUC reduction for the combination compared to 2.3% and 4.3% for the respective monotherapies (at ×0.01 blockade). The combination effect was sub-additive rather than synergistic when tested against the Bliss independence model (observed 5.2% vs Bliss-expected 6.5%). A factorial NAC design encompassing control, anti-TLR4, anti-IL-17, and combination arms, with TNSS and cytokine measurements under each condition, would provide rigorous validation of this combination prediction. Collectively, H2, H3, and H5 remain predicted but untested, awaiting appropriate clinical NAC study data, while H1 and H4 have received partial support from model simulations and existing literature.

### 4.3 Limitations

Several limitations should be acknowledged:

1. Over half of the model parameters (51%) remain estimated rather than directly measured, and absolute calibration relies on pg/mL values approximated from published figures (Linton 2025 (16), Campion 2023 (25)). However, Sobol sensitivity analysis provides reassurance that the most influential parameters (k_S1, k_Med1, k_S5, k_S2) belong to the well-characterized core Th2/IgE axis, while estimated new-pathway parameters carry low sensitivity indices, limiting their impact on model behavior.
2. Clinical validation is partial and drug mechanisms are simplified. Dupilumab AUC prediction is reasonable (within 6 percentage points of trial data), but peak reduction and intranasal corticosteroid symptom reduction are underestimated, reflecting the model’s parameter-scaling approach versus real pharmacological effects such as mast cell depletion and vascular changes. The seasonal tolerance mechanism also underestimates the early-late symptom difference (-0.07 vs de Weger’s -0.30 to -0.41), indicating the tolerance state variable saturates too slowly.
3. The virtual cohort is entirely synthetic. The 2,000-simulated-individual virtual cohort uses lognormal parameter distributions (CV = 30%) without real population data, and the three identified subtypes are mathematical constructs that require validation against real patient heterogeneity. The subtype proportions (40%/38%/22%) reflect sampling assumptions and may not match real prevalence. Independent parameter sampling fails to capture the biological covariation present in real patients, contributing to weak absolute H4 correlations (ρ ≈ 0.08).
4. The Th17/Treg mutual antagonism is modeled with linear terms rather than mechanistic RORγt/Foxp3 transcription factor dynamics, which may limit the model’s ability to capture regulatory T cell plasticity and the nonlinear feedback loops that govern Th17/Treg fate decisions *in vivo*.
5. Formal parameter identifiability analysis was not performed. With 57 parameters in the 19-dimensional model and limited calibration data (11 published studies), many parameters are likely practically non-identifiable. Sobol sensitivity analysis provides partial reassurance that the most influential parameters (k_S1, k_Med1, k_S5, k_S2) belong to the well-characterized core axis, while estimated new-pathway parameters carry low sensitivity indices. Future work should employ multi-start optimization and profile-likelihood analysis to assess identifiability.
6. A non-negativity verification was performed on all 21 state variables. Under standard parameters, all variables remain non-negative throughout the simulation. In the virtual cohort, however, the symptom score (Sym) can reach −0.48 under extreme IL-10 suppression, an artifact of the linear suppression term. This was addressed by clamping Sym to (0, 10) in post-processing; the affected cases constitute < 3% of the cohort and do not alter the clustering or sensitivity results (**Supplementary Table S22**).

### 4.4 Future directions

Several extensions are envisioned to address the limitations identified above. First, incorporating RORγt/Foxp3 transcription factor dynamics would provide a more mechanistic representation of Th17/Treg plasticity and mutual antagonism. Second, calibrating the virtual cohort against real patient data—serum IgE, nasal eosinophil counts, and longitudinal symptom diaries—would enable personalized treatment prediction and resolve the subtype proportion and H4 correlation discrepancies. Third, coupling the model with pharmacokinetic frameworks would allow dose-response simulation for oral antihistamines and subcutaneous biologics, moving beyond the current parameter-scaling drug representation. Fourth, adding vascular permeability and mucosal edema state variables would improve INCS symptom prediction by capturing glucocorticoid effects on nasal congestion that are independent of mast cell degranulation. Finally, the remaining untested hypotheses (H2, H3, H5) warrant validation through targeted NAC studies employing TLR4 antagonists, low-dose IL-2, and dual-blockade factorial designs.

## 5 Conclusion

We present a progressively extended ODE model of seasonal allergic rhinitis that evolves from a 10-dimensional core Th2/IgE/mast cell/eosinophil axis to a 21-dimensional system integrating three immunological pathways (core Th2 axis, TLR4/NF-κB innate immunity, Th17/Treg imbalance) with two dynamic processes (priming and tolerance) across two temporal scales (acute 24h NAC and chronic 8-week season). The model is calibrated against absolute cytokine concentrations, validated against clinical trial drug efficacy, and its predictions regarding Th17/Treg imbalance as a late-phase severity marker are directionally consistent with published clinical data. AI analysis of a 2,000-patient virtual cohort identifies three virtual subtypes mapping to published AR phenotypes. The model generates five testable hypotheses with specific validation approaches, positioning it as a hypothesis-generating tool rather than a predictive clinical model. The finding that 22% of simulated individuals in the virtual cohort exhibit a severe IL-17-high phenotype supports the hypothesis that Th17/Treg-targeted therapies may benefit a biologically defined subgroup of SAR patients who respond inadequately to standard Th2-focused treatments, warranting validation in real patient cohorts.

## Data Availability

The original contributions presented in the study are included in the article/**Supplementary Material**. Further inquiries can be directed to the corresponding author.
