# Supplementary material for: A progressive ODE model of seasonal allergic rhinitis: from core Th2 axis to 21-dimensional multi- pathway integration with seasonal dynamics and directional assessment
Source: Front Immunol. 2026 Oct 29;17:1971520. doi: 10.3389/fimmu.2026.1971520 (PMC13754829; doi:10.3389/fimmu.2026.1971520)
Supplement: Supplementary file 1 [file Table1.docx]

Supplementary Material

A Progressive ODE Model of Seasonal Allergic Rhinitis: From Core Th2 Axis to 21-Dimensional Multi-Pathway Integration with Seasonal Dynamics and Directional Assessment

## Supplementary Table S1. Core 10-dimensional model parameters (26 total).

| **parameter** | **value** | **unit** | **source** | **note** |
| --- | --- | --- | --- | --- |
| k_A | 1.4 | /h | literature | Mucociliary clearance t1/2 ~ 30 min |
| k_Al1 | 2.0 | /h | estimated | Epithelial rapid alarmin response |
| k_Al2 | 1.0 | /h | estimated | IL-33 degradation t1/2 ~ 40 min |
| Al_max | 500.0 | pg/mL | estimated | Local alarmin concentration ceiling |
| k_DC1 | 0.5 | /h | estimated | DC activation by alarmins |
| k_DC2 | 0.12 | /h | literature | DC tissue half-life ~ 6 h |
| DC_max | 50.0 | cells/uL | estimated | Max activated DC in nasal mucosa |
| k_T1 | 0.3 | /h | estimated | Th2 activation by DC antigen presentation |
| k_T2 | 0.06 | /h | literature | Effector Th2 half-life ~ 12 h |
| Th2_max | 80.0 | cells/uL | estimated | Max Th2 proliferation |
| k_C1 | 8.0 | pg/(mL*h*cell) | estimated | Th2 cytokine (IL-4/5/13) production rate |
| k_C2 | 0.7 | /h | literature | IL-4 degradation t1/2 ~ 60 min |
| k_IgE0 | 2.1 | ng/(mL*h) | calculated | Baseline IgE production (maintains IgE_ss=150) |
| k_IgE1 | 0.005 | /h | estimated | Cytokine-driven IgE upregulation |
| k_IgE2 | 0.014 | /h | literature | IgE half-life ~ 2.5 days |
| k_MC1 | 0.0008 | /h | estimated | Fc-epsilon-RI cross-linking efficiency |
| k_MC2 | 1.4 | /h | literature | Mast cell degranulation recovery ~ 30 min |
| k_Med1 | 200.0 | ng/(mL*h) | estimated | Mediator (histamine+LT) release rate |
| k_Med2 | 4.0 | /h | literature | Histamine clearance t1/2 ~ 10 min |
| k_Eos1 | 0.5 | cells/(uL*h*pg/mL) | estimated | IL-5-driven eosinophil recruitment |
| k_Eos2 | 0.15 | /h | literature-adjusted | Tissue eosinophil survival ~ 5 h |
| Eos_max | 200.0 | cells/uL | estimated | Max tissue eosinophil burden |
| k_S1 | 0.4 | (score*mL)/(ng*h) | calibrated | Mediator-to-symptom coefficient (immediate phase) |
| k_S2 | 0.015 | score/(cell*h) | calibrated | Eosinophil-to-symptom coefficient (late phase) |
| k_S5 | 0.5 | /h | estimated | Symptom resolution t1/2 ~ 1.4 h |
| Sym_max | 10.0 | score | fixed | Maximum symptom score (VAS scale) |

## Supplementary Table S2. Extended model state variables (19 total).

| **Variable** | **Description** | **Unit** | **Initial Condition** | **Biological Basis** |
| --- | --- | --- | --- | --- |
| A | Allergen concentration | ng/mL | 100 | Single pollen exposure bolus |
| Al | Epithelial alarmins (TSLP+IL-33) | pg/mL | 0 | No pre-existing damage |
| DC | Activated dendritic cells | cells/µL | 5 | Baseline mucosal DC |
| Th2 | Activated Th2 cells | cells/µL | 2 | Memory Th2 (sensitized) |
| Cyto | Th2 cytokines (IL-4/5/13) | pg/mL | 0 | No acute inflammation |
| IgE | Allergen-specific IgE | ng/mL | 150 | Pre-existing IgE (sensitized) |
| MC | Mast cell activation fraction | 0-1 | 0 | Not activated |
| Med | Inflammatory mediators | ng/mL | 0 | Not released |
| Eos | Nasal eosinophils | cells/µL | 10 | Baseline few |
| Sym | Symptom score | 0-10 | 0 | Asymptomatic |
| TLR4 | TLR4 activation | 0-1 | 0 | Not activated |
| MyD88 | MyD88 recruitment | 0-1 | 0 | Not recruited |
| NFkB | NF-κB activation | 0-1 | 0 | Cytoplasmic (inactive) |
| ProInf | Pro-inflammatory cytokines | pg/mL | 0 | No acute inflammation |
| Neu | Neutrophils | cells/µL | 5 | Baseline circulating |
| Th17 | Th17 cells | cells/µL | 1 | Baseline low |
| IL17 | IL-17 | pg/mL | 0 | No acute inflammation |
| Treg | Regulatory T cells | cells/µL | 5 | Baseline regulatory |
| IL10 | IL-10 | pg/mL | 2 | Baseline low |

## Supplementary Table S3. Extended model new parameters (TLR4 + Th17/Treg pathways).

| **Parameter** | **Description** | **Value** | **Unit** | **Pathway** | **Source** | **Source_detail** |
| --- | --- | --- | --- | --- | --- | --- |
| k_A | Allergen clearance rate | 1.4 | /h | Core axis | Literature | Literature: t1/2 ~ 30 min |
| k_Al1 | Alarmin production rate | 2.0 | /h | Core axis | Estimated | Estimated: epithelial rapid response |
| k_Al2 | Alarmin degradation rate | 1.0 | /h | Core axis | Estimated | Estimated: IL-33 t1/2 ~ 40 min |
| Al_max | Alarmin concentration ceiling | 500.0 | pg/mL | Core axis | Estimated | Estimated |
| k_DC1 | DC activation by alarmins | 0.5 | /h | Core axis | Estimated | Estimated |
| k_DC2 | DC tissue decay rate | 0.12 | /h | Core axis | Literature | Literature: t1/2 ~ 6h |
| DC_max | Max activated DC | 50.0 | cells/μL | Core axis | Estimated | Estimated |
| k_T1 | Th2 activation by DC | 0.3 | /h | Core axis | Estimated | Estimated |
| k_T2 | Th2 effector decay | 0.06 | /h | Core axis | Literature | Literature: t1/2 ~ 12h |
| Th2_max | Max Th2 cells | 80.0 | cells/μL | Core axis | Estimated | Estimated |
| k_C1 | Th2 cytokine production | 8.0 | pg/(mL·h·cell) | Core axis | Estimated | Estimated |
| k_C2 | Cytokine degradation | 0.7 | /h | Core axis | Literature | Literature: IL-4 t1/2 ~ 60 min |
| k_IgE0 | Baseline IgE production | 2.1 | ng/(mL·h) | Core axis | Calculated | Calculated: maintains IgE_ss=150 |
| k_IgE1 | Cytokine-driven IgE upregulation | 0.005 | /h | Core axis | Calibrated | Estimated (calibrated) |
| k_IgE2 | IgE degradation | 0.014 | /h | Core axis | Literature | Literature: t1/2 ~ 2.5 days |
| k_MC1 | FcεRI cross-linking efficiency | 0.0008 | /h | Core axis | Estimated | Estimated |
| k_MC2 | Mast cell recovery | 1.4 | /h | Core axis | Literature | Literature: ~ 30 min |
| k_Med1 | Mediator release rate | 200.0 | ng/(mL·h) | Core axis | Calibrated | Estimated (calibrated) |
| k_Med2 | Mediator clearance | 4.0 | /h | Core axis | Literature | Literature: histamine t1/2 ~ 10 min |
| k_Eos1 | Eosinophil recruitment | 0.5 | cells/(μL·h·pg/mL) | Core axis | Estimated | Estimated |
| k_Eos2 | Eosinophil apoptosis | 0.15 | /h | Core axis | Literature | Literature-adjusted: t1/2 ~ 5h |
| Eos_max | Max eosinophil burden | 200.0 | cells/μL | Core axis | Estimated | Estimated |
| k_S1 | Mediator→symptom sensitivity | 0.4 | (score·mL)/(ng·h) | Core axis | Calibrated | Calibrated |
| k_S2 | Eosinophil→symptom sensitivity | 0.015 | score/(cell·h) | Core axis | Calibrated | Calibrated |
| k_S5 | Symptom resolution rate | 0.5 | /h | Core axis | Estimated | Estimated: t1/2 ~ 1.4h |
| Sym_max | Max symptom score | 10.0 | score | Core axis | Fixed | Fixed |
| k_TLR1 | TLR4 activation by allergen | 1.5 | /h | TLR4/MyD88/NF-κB | Estimated | Estimated: pollen rapid activation |
| k_TLR2 | TLR4 deactivation | 0.7 | /h | TLR4/MyD88/NF-κB | Estimated | Estimated: t1/2 ~ 1h |
| k_MyD1 | MyD88 recruitment | 3.0 | /h | TLR4/MyD88/NF-κB | Estimated | Estimated: fast cascade |
| k_MyD2 | MyD88 degradation | 1.5 | /h | TLR4/MyD88/NF-κB | Estimated | Estimated |
| k_NF1 | NF-κB activation | 2.5 | /h | TLR4/MyD88/NF-κB | Estimated | Estimated: rapid nuclear translocation |
| k_NF2 | NF-κB inhibition (IκB feedback) | 1.0 | /h | TLR4/MyD88/NF-κB | Estimated | Estimated |
| k_PI1 | Pro-inflammatory cytokine production | 15.0 | pg/(mL·h) | TLR4/MyD88/NF-κB | Estimated | Estimated |
| k_PI2 | Pro-inflammatory cytokine degradation | 0.5 | /h | TLR4/MyD88/NF-κB | Literature | Literature: TNF-α t1/2 ~ 1.4h |
| k_Neu1 | Neutrophil recruitment by pro-inf | 0.3 | cells/(μL·h·pg/mL) | TLR4/MyD88/NF-κB | Estimated | Estimated |
| k_Neu2 | Neutrophil apoptosis | 0.2 | /h | TLR4/MyD88/NF-κB | Literature | Literature: tissue survival ~ 3.5h |
| Neu_max | Max neutrophils | 150.0 | cells/μL | TLR4/MyD88/NF-κB | Estimated | Estimated |
| k_AlNF | NF-κB→alarmin enhancement | 50.0 | pg/(mL·h) | TLR4/MyD88/NF-κB | Estimated | Estimated |
| k_DCNF | NF-κB→DC enhancement | 0.2 | /h | TLR4/MyD88/NF-κB | Estimated | Estimated |
| k_Th17_1 | Th17 differentiation rate | 0.005 | /h | Th17/Treg | Estimated | Estimated: requires DC+IL-6 |
| k_Th17_2 | Th17 decay | 0.04 | /h | Th17/Treg | Literature | Literature: t1/2 ~ 17h |
| Th17_max | Max Th17 cells | 30.0 | cells/μL | Th17/Treg | Estimated | Estimated |
| k_IL17_1 | IL-17 production | 5.0 | pg/(mL·h·cell) | Th17/Treg | Estimated | Estimated |
| k_IL17_2 | IL-17 degradation | 0.5 | /h | Th17/Treg | Literature | Literature: t1/2 ~ 1.4h |
| k_Treg_1 | Treg expansion | 0.02 | /h | Th17/Treg | Estimated | Estimated |
| k_Treg_2 | Treg decay | 0.02 | /h | Th17/Treg | Estimated | Estimated: steady-state maintenance |
| Treg_max | Max Treg cells | 20.0 | cells/μL | Th17/Treg | Estimated | Estimated |
| k_IL10_1 | IL-10 production | 3.0 | pg/(mL·h·cell) | Th17/Treg | Estimated | Estimated |
| k_IL10_2 | IL-10 degradation | 0.3 | /h | Th17/Treg | Literature | Literature: t1/2 ~ 2.3h |
| k_Treg_inh | Treg suppresses Th17 | 0.01 | /h | Th17/Treg | Estimated | Estimated |
| k_Th17_inh | Th17 suppresses Treg | 0.005 | /h | Th17/Treg | Estimated | Estimated |
| k_T1IL17 | IL-17 enhances Th2 | 0.002 | /h | Th17/Treg | Estimated | Estimated |
| k_Neu1IL17 | IL-17 recruits neutrophils | 0.1 | cells/(μL·h·pg/mL) | Th17/Treg | Estimated | Estimated |
| k_IL10_DC | IL-10 suppresses DC | 0.001 | /h | Th17/Treg | Estimated | Estimated |
| k_S3b | Neutrophil→symptom | 0.008 | score/(cell·h) | Cross-connection | Calibrated | Calibrated |
| k_S4 | Pro-inflammatory→symptom | 0.003 | (score·mL)/(pg·h) | Cross-connection | Calibrated | Calibrated |
| k_S_IL10 | IL-10 suppresses symptom | 0.01 | (score·mL)/(pg·h) | Cross-connection | Calibrated | Calibrated |

## Supplementary Table S4. Quantitative dynamics of all 10 core model variables.

| **Variable** | **Peak Value** | **Peak Time (h)** | **Late Value (12h)** | **AUC (0-24h)** |
| --- | --- | --- | --- | --- |
| A (Allergen) | 100.0 | 0 | 0.5 | 41.2 |
| Al (Alarmins) | 285.0 | 0.5 | 12.0 | 890.0 |
| DC | 47.0 | 1.0 | 8.0 | 210.0 |
| Th2 | 38.0 | 2.0 | 15.0 | 180.0 |
| Cyto (IL-4/5/13) | 340.0 | 2.5 | 45.0 | 1200.0 |
| IgE | 185.0 | 4.0 | 165.0 | 3200.0 |
| MC (fraction) | 0.84 | 0.4 | 0.02 | 3.2 |
| Med | 47.6 | 0.9 | 3.0 | 180.0 |
| Eos | 174.0 | 14 | 120.0 | 1850.0 |
| Sym | 7.0 | 1.9 | 3.4 | 93.3 |

## Supplementary Table S5. Sobol sensitivity indices — top 5 parameters.

| **Parameter** | **S1_Sym_max** | **ST_Sym_max** | **S1_AUC** | **ST_AUC** |
| --- | --- | --- | --- | --- |
| k_S1 | 0.3771284222601397 | 0.3987455223225163 | 0.0198495748007904 | 0.0252289821210211 |
| k_Med1 | 0.3869154977709931 | 0.3962069077451835 | 0.02086314376802 | 0.0247312590404452 |
| k_S5 | 0.1593974695228676 | 0.1537794801581218 | 0.3276122671915064 | 0.305655310901654 |
| k_A | 0.0388205683516032 | 0.0369759349981527 | 0.0499856700213889 | 0.0452419350748868 |
| k_MC1 | 0.0191415959744241 | 0.0294158144002146 | 0.0025642717878794 | 0.004484940059767 |
| k_S2 | -0.007958069743732 | 0.0018436377714501 | 0.3403438075414104 | 0.3533695753924141 |
| k_S_IL10 | 0.0027402155827589 | 0.001434593328538 | 0.1045577560959174 | 0.1283912207713015 |
| k_IL10_1 | -0.0025021692773044 | 0.0012406572660264 | 0.0717030426123175 | 0.0869440336438776 |
| k_Eos1 | 0.0006751761261186 | 0.00027756563257 | 0.0135949568001806 | 0.008988188171451 |
| k_C1 | -2.1682045006107456e-05 | 0.0002637156842772 | 0.0063183863205028 | 0.0097653249822702 |
| k_T1 | -9.928068534211032e-05 | 0.0001072206666544 | 0.0057581889805419 | 0.0038591584935711 |
| k_DC1 | 0.0007109892844297 | 3.695131095895927e-05 | -0.001703312118039 | 0.001484821557873 |
| k_PI1 | -0.0001713528672166 | 2.002701114301792e-05 | 0.0028713557619659 | 0.0029967765578201 |
| k_Treg_1 | 0.0001814972906439 | 1.405099897426136e-05 | 0.0194809362096217 | 0.0224010041408961 |
| k_Al1 | 0.0003772640080485 | 1.2644109603011618e-05 | -0.0019037756144421 | 0.0001207046322539 |
| k_NF1 | -5.925218567675221e-05 | 3.074766793897401e-06 | 0.0004713800672519 | 0.0003791380853391 |
| k_Neu1 | -2.8497920853874404e-05 | 7.048417815555287e-07 | 0.0002924974058887 | 0.0001544941493572 |
| k_IL17_1 | 3.110260543935573e-05 | 3.3614351029230346e-07 | 0.0020010819615399 | 0.0014665499360472 |
| k_Th17_1 | -3.925544120423756e-05 | 2.476654599934506e-07 | 0.0014639589097574 | 0.001510364362607 |
| k_IgE1 | 6.729005551238481e-06 | 1.1756818604928e-08 | -7.200939898181041e-05 | 1.2538600113699716e-06 |
| k_TLR1 | 2.985338986283485e-06 | 1.0385014445656876e-09 | -0.0001222615213031 | 3.835220189855043e-06 |

## Supplementary Table S6. Subtype-specific drug response (% AUC reduction).

*Subtype-specific drug responses computed using PCA-reduced K-means subtypes (PC1+PC2, 57.7% cumulative variance). K=3, silhouette=0.36, ARI=0.98.*

| **Subtype** | **Drug** | **AUC** | **Sym_max** | **AUC_reduction_pct** |
| --- | --- | --- | --- | --- |
| Subtype 1 | Control | 92.18468866240804 | 7.301456858516813 | 0.0 |
| Subtype 1 | Anti-IL-17 (secukinumab) | 89.72098637455673 | 7.300534399265012 | 2.785963212293552 |
| Subtype 1 | Dupilumab (anti-IL-4Rα) | 73.85255437402087 | 7.27055825369864 | 20.51962382761914 |
| Subtype 1 | Anti-TLR4 (antagonist) | 91.63949988391562 | 7.300774065915852 | 0.6143991586047878 |
| Subtype 1 | Treg enhancement (low-dose IL-2) | 76.93547671065734 | 7.20933939642492 | 17.52203385317877 |
| Subtype 2 | Control | 64.28013327088026 | 5.641567097240248 | 0.0 |
| Subtype 2 | Anti-IL-17 (secukinumab) | 61.67937772276586 | 5.640070123138218 | 4.35989206600191 |
| Subtype 2 | Dupilumab (anti-IL-4Rα) | 46.71633336223964 | 5.605904756999012 | 28.122210984592 |
| Subtype 2 | Anti-TLR4 (antagonist) | 63.69175956913213 | 5.640599089267352 | 0.9724703763264472 |
| Subtype 2 | Treg enhancement (low-dose IL-2) | 49.22037380591185 | 5.52583777063931 | 25.10651958974688 |
| Subtype 3 | Control | 89.4356197657928 | 6.862513424979154 | 0.0 |
| Subtype 3 | Anti-IL-17 (secukinumab) | 84.707144198205 | 6.858993683555589 | 5.638840195708358 |
| Subtype 3 | Dupilumab (anti-IL-4Rα) | 73.06827755900235 | 6.8243692998529575 | 19.135087313589423 |
| Subtype 3 | Anti-TLR4 (antagonist) | 88.79931804931265 | 6.861370746374668 | 0.7478411717476783 |
| Subtype 3 | Treg enhancement (low-dose IL-2) | 74.59017159294964 | 6.756517335103395 | 17.782996626869085 |

## Supplementary Table S7. PCA-reduced K-means virtual subtypes.

*Three subtypes identified by K-means on first two principal components of six standardized output metrics. PC1 (32.9%): Th17/neutrophil axis. PC2 (24.8%): Th2-eosinophilic burden. Cluster sizes: 793 (40%), 757 (38%), 450 (22%).*

| **Subtype** | **N** | **Proportion** | **Sym_max_mean** | **AUC_mean** | **Eos_peak_mean** | **Neu_peak_mean** | **IL17_peak_mean** |
| --- | --- | --- | --- | --- | --- | --- | --- |
| 1 | 793 | 40% | 7.306403918450241 | 94.88789640706092 | 195.00016297079304 | 34.74298405650717 | 87.30973695199992 |
| 2 | 757 | 38% | 5.638633189462726 | 58.51470337498184 | 148.72957700705928 | 35.569467254564685 | 91.75584643633395 |
| 3 | 450 | 22% | 6.865698339472964 | 88.25375586650956 | 174.79444919307562 | 68.91940435148321 | 227.22604517286663 |

## Supplementary Table S8. Fold-change calibrated parameters.

| **Parameter** | **Original** | **Calibrated** | **Change_pct** |
| --- | --- | --- | --- |
| k_PI1 | 15.0 | 4.5 | -70.0 |
| k_Th17_1 | 0.005 | 0.0015 | -70.0 |
| k_IL17_1 | 5.0 | 4.701245417397597 | -5.975091652048068 |
| k_IL10_1 | 3.0 | 8.63469010527861 | 187.823003509287 |
| k_AlNF | 50.0 | 48.34999420067464 | -3.300011598650712 |
| k_C1 | 8.0 | 2.4 | -70.0 |

## Supplementary Table S9. Absolute calibration scaling factors.

| **Parameter** | **Initial** | **Calibrated** | **Change_pct** |
| --- | --- | --- | --- |
| scale_Cyto | 3.5 | 0.3706368162488627 | -89.41037667860392 |
| scale_ProInf | 4.0 | 8.56663476478345 | 114.16586911958623 |
| scale_IL17 | 12.0 | 12.0 | 0.0 |
| scale_IL10 | 2.0 | 2.0 | 0.0 |
| scale_Eos | 0.2 | 0.451688164084928 | 125.844082042464 |
| k_C1 | 8.0 | 0.8 | -90.0 |
| k_PI1 | 15.0 | 6.3060801467418415 | -57.95946568838772 |

## Supplementary Table S10. Absolute calibration quality (RMSE).

| **Cytokine** | **RMSE_pgmL** | **model_peak** | **literature_peak** | **peak_ratio** |
| --- | --- | --- | --- | --- |
| IL-5 | 4.209493914568849 | 14.890053496534827 | 18.0 | 0.8272251942519349 |
| IL-13 | 2.6966448533831686 | 10.423037447574378 | 12.0 | 0.8685864539645315 |
| IL-6 | 17.580782887837227 | 57.91381353751381 | 55.0 | 1.0529784279547965 |
| TNF-α | 7.815202018641983 | 28.956906768756905 | 25.0 | 1.1582762707502765 |
| Eosinophils | 11.987994662853712 | 38.40070933098348 | 35.0 | 1.0971631237423851 |

## Supplementary Table S11. Absolute calibration scaling factors (detailed).

| **Cytokine** | **Source Variable** | **Splitting Factor** | **Baseline (pg/mL)** | **Post-Calibration (pg/mL)** |
| --- | --- | --- | --- | --- |
| IL-4 | Cyto | 0.40 | 136.0 | 97.2 |
| IL-5 | Cyto | 0.35 | 119.0 | 85.1 |
| IL-13 | Cyto | 0.25 | 85.0 | 60.8 |
| TNF-α | ProInf | 0.45 | 7.3 | 5.2 |
| IL-6 | ProInf | 0.35 | 5.7 | 4.1 |
| IL-1β | ProInf | 0.20 | 3.2 | 2.3 |

## Supplementary Table S12. Clinical trial validation results.

| **Drug** | **Endpoint** | **Time Window** | **Comparison Type** | **Model Prediction** | **Clinical Value** | **Gap** | **Source** |
| --- | --- | --- | --- | --- | --- | --- | --- |
| Dupilumab | TNSS AUC reduction | 0-1h post-NAC | Between-group (SCIT+dupilumab vs placebo) | 27.8% | 33.9% | 6.1 pp | Corren 2021 [26], n=103 |
| Dupilumab | Peak TNSS reduction | 0-1h post-NAC | Between-group (SCIT+dupilumab vs placebo) | 0.3% | 24.4% | 24.1 pp | Corren 2021 [26], n=103 |
| INCS (triamcinolone) | IL-13 reduction | 7h post-NAC | Between-group (INCS vs placebo) | 93.0% | 93.0% | 0.0 pp (calibration) | Bauer 2023 [24], n=20 |
| INCS (triamcinolone) | TNSS AUC reduction | 0-30min post-NAC | Between-group (INCS vs placebo) | 27.2% | 32.0% | 4.8 pp | Bauer 2023 [24], n=20 |

## Supplementary Table S13. Seasonal extension parameters.

| **Parameter** | **Description** | **Value** | **Unit** | **Source** |
| --- | --- | --- | --- | --- |
| k_Prime1 | Priming accumulation rate | 0.08 | /h | Estimated |
| k_Prime2 | Priming decay rate | 0.002 | /h | Estimated (t½ ~6 days) |
| k_Prime_amp | Priming amplification factor | 2.0 | — | Estimated |
| k_Tol1 | Tolerance accumulation rate | 0.003 | /h | Estimated |
| k_Tol2 | Tolerance decay rate | 0.0002 | /h | Estimated (t½ ~60 days) |
| k_Tol_damp | Tolerance dampening factor | 0.5 | — | Estimated |
| Pollen_peak_day | Gaussian seasonal peak | 21 | day | Estimated |
| Pollen_spread | Gaussian spread | 14 | days | Estimated |

## Supplementary Table S14. Repetitive NAC priming data.

| **Challenge** | **Sym_peak** | **IL5_peak_pgmL** | **Eos_peak** |
| --- | --- | --- | --- |
| 1 | 6.9029544317496745 | 189.89426722718025 | 178.99581987195558 |
| 2 | 6.911423095189998 | 225.4853282275756 | 182.0248636369141 |
| 3 | 6.921479721086482 | 249.30727599248232 | 183.60667202930455 |
| 4 | 6.932573911043492 | 265.6965521644561 | 184.54275756208884 |
| 5 | 6.944252552196653 | 277.39396744761353 | 185.14791634197837 |

## Supplementary Table S15. Subtype-phenotype mapping.

| **Subtype** | **N** | **Proportion** | **Key Features** | **Mapped Phenotype** | **Reference** |
| --- | --- | --- | --- | --- | --- |
| Subtype 1 | 793 | 40% | High-symptom Th2-eosinophilic: Sym_max 7.3, AUC 94.9, Eos_peak 195, IL-17 87 (low), Neu 35 | Th2-dominant moderate-severe AR | Cai 2024 [29] |
| Subtype 2 | 757 | 38% | Mild low-inflammation: Sym_max 5.6, AUC 58.5, Eos_peak 149, IL-17 92, Neu 36 | Mild AR / low-type-2 inflammation | Campion 2023 [25] |
| Subtype 3 | 450 | 22% | Th17-high: IL-17 227 (highest), Neu 69 (highest), Sym_max 6.9, AUC 88.3 | Th17-high severe AR | Xiong 2026 [12], Gu 2017 [13] |

## Supplementary Table S16. H4 validation results.

| **Metric** | **Value** | **P_value** |
| --- | --- | --- |
| Spearman ρ (Th17/Treg vs late AUC) | 0.0790037117509279 | 0.0004055077127136 |
| Spearman ρ (IgE vs late AUC) | 0.025304808326202 | 0.2579965684580938 |
| Spearman ρ (Th17/Treg vs total AUC) | 0.0712599238149809 | 0.0014281388884014 |
| Spearman ρ (IgE vs total AUC) | 0.0828655512163877 | 0.0002073205856337 |
| ROC AUC (Th17/Treg alone) | 0.529785 |  |
| ROC AUC (IgE alone) | 0.51868 |  |
| ROC AUC (Combined) | 0.53834 |  |
| H4 supported | 1.0 |  |

## Supplementary Table S17. Ablation analysis: pathway contributions to symptom burden.

| **Model_variant** | **Sym_max** | **t_peak** | **AUC_0_24h** | **AUC_late_6_24h** | **AUC_immediate_0_2h** |
| --- | --- | --- | --- | --- | --- |
| Core (10-dim) | 6.990341797080902 | 1.9 | 93.34701232735956 | 61.86492509750573 | 9.570914015159303 |
| Full (19-dim) | 6.903962714898128 | 1.85 | 82.7162239583817 | 52.27895679045531 | 9.496765993734702 |
| Core+TLR4 (15-dim) | 7.001272335643986 | 1.9 | 96.46252291671269 | 64.72621380369745 | 9.591180869330008 |
| Core+Th17/Treg (14-dim) | 6.892576760381608 | 1.85 | 77.1372967748567 | 46.99376662936884 | 9.475975653034087 |

## Supplementary Table S18. Sobol convergence check: N=256 vs N=512.

| **N** | **Parameter** | **S1** | **S1_conf** | **ST** | **ST_conf** |
| --- | --- | --- | --- | --- | --- |
| 256 | k_S1 | 0.0370878540887366 | 0.0341990389447003 | 0.0339902236830933 | 0.0062429631787542 |
| 256 | k_Med1 | 0.0376100192283387 | 0.0360987153119656 | 0.0345074179924328 | 0.0071028067273562 |
| 256 | k_S2 | 0.4171658403070369 | 0.1055603537593061 | 0.4238926775966394 | 0.0838374865414842 |
| 256 | k_S5 | 0.4570003221320424 | 0.1046131362445042 | 0.4581709047106336 | 0.0796154166573055 |
| 256 | k_S_IL10 | 0.0650373588897849 | 0.0454465997621996 | 0.0681835016215267 | 0.0119641020487587 |
| 512 | k_S1 | 0.0350345973117397 | 0.0231517335443889 | 0.034570737845718 | 0.0041450878101719 |
| 512 | k_Med1 | 0.0346966215037825 | 0.0240111412669952 | 0.0339645479418855 | 0.0042636802488986 |
| 512 | k_S2 | 0.4167944452125568 | 0.0752084917700566 | 0.4214420979612426 | 0.0539156184969032 |
| 512 | k_S5 | 0.4444901179227854 | 0.0733133934851301 | 0.4534068362198522 | 0.0537449797147844 |
| 512 | k_S_IL10 | 0.065059833446157 | 0.0289101645169288 | 0.0683355419913704 | 0.010238532958356 |

## Supplementary Table S19. Drug-effect parametric sweep: anti-TLR4 and anti-IL-17 at multiple blockade levels.

| **Drug** | **Blockade_factor** | **AUC** | **Peak** | **AUC_reduction_pct** | **Peak_reduction_pct** |
| --- | --- | --- | --- | --- | --- |
| Anti-TLR4 | 0.01 | 80.83826981234151 | 6.901623508839214 | 2.2703576833791206 | 0.0338820783876259 |
| Anti-IL-17 | 0.01 | 79.17823150853036 | 6.9034670757927215 | 4.277265426951149 | 0.0071790524641346 |
| Anti-TLR4 | 0.05 | 81.87059349960134 | 6.903183710016098 | 1.0223272029509456 | 0.0112834456702626 |
| Anti-IL-17 | 0.05 | 79.35611021532691 | 6.903487131460098 | 4.062218489017855 | 0.0068885574512678 |
| Anti-TLR4 | 0.1 | 82.12492754826333 | 6.903552452692139 | 0.7148493751550911 | 0.00594241630395 |
| Anti-IL-17 | 0.1 | 79.5734052102082 | 6.90351219750432 | 3.799519124270956 | 0.0065254899600852 |
| Anti-TLR4 | 0.2 | 82.33392122409913 | 6.903768492226661 | 0.4621859122521443 | 0.002813205683283 |
| Anti-IL-17 | 0.2 | 79.99191451833647 | 6.903562317803739 | 3.2935611778119323 | 0.0057995257350618 |
| Anti-TLR4 | 0.5 | 82.56525574197873 | 6.903911997560551 | 0.1825134286581264 | 0.0007346119854978 |
| Anti-IL-17 | 0.5 | 81.13175071161115 | 6.903712584426989 | 1.9155531659276197 | 0.0036229985802105 |
| Combination (Anti-TLR4 + Anti-IL-17) | 0.01 | 78.395314330897 | 6.901140231925245 | 5.223775241068496 | 0.0408820714919655 |
| Combination (Anti-TLR4 + Anti-IL-17) | 0.05 | 78.93160855448494 | 6.902713105964323 | 4.575420906303673 | 0.0180998795243914 |
| Combination (Anti-TLR4 + Anti-IL-17) | 0.1 | 79.2421281369196 | 6.903104555115738 | 4.200017427306735 | 0.0124299596887755 |
| Combination (Anti-TLR4 + Anti-IL-17) | 0.2 | 79.74365581550092 | 6.9033692254887775 | 3.5936942000355576 | 0.0085963588428716 |
| Combination (Anti-TLR4 + Anti-IL-17) | 0.5 | 81.00588458685294 | 6.903662053751206 | 2.067719353810582 | 0.0043549068750592 |

## Supplementary Table S20. Bliss independence synergy test for anti-TLR4 + anti-IL-17 combination.

| **Blockade_factor** | **E_TLR4** | **E_IL17** | **E_observed** | **E_bliss_expected** | **Bliss_excess** | **Synergistic** |
| --- | --- | --- | --- | --- | --- | --- |
| 0.01 | 2.2703576833791206 | 4.277265426951149 | 5.223775241068496 | 6.450513886070966 | -1.2267386450024702 | False |
| 0.05 | 1.0223272029509456 | 4.062218489017855 | 4.575420906303673 | 5.043016527312268 | -0.4675956210085951 | False |
| 0.1 | 0.7148493751550911 | 3.799519124270956 | 4.200017427306735 | 4.4872076607072975 | -0.2871902334005631 | False |
| 0.2 | 0.4621859122521443 | 3.2935611778119323 | 3.5936942000355576 | 3.740524714288824 | -0.1468305142532662 | False |
| 0.5 | 0.1825134286581264 | 1.9155531659276197 | 2.067719353810582 | 2.094570452824842 | -0.0268510990142599 | False |

## Supplementary Table S21. K-means stability analysis using PCA-reduced output metrics across K values and random seeds.

*K-means on first two principal components of six standardized output metrics. K=3 selected: silhouette=0.36 (highest), ARI=0.98 (range 0.95–1.00), bootstrap ARI=0.94±0.04.*

| **K** | **Seed** | **Silhouette** | **Cluster_sizes** | **Min_cluster** | **Max_cluster** |
| --- | --- | --- | --- | --- | --- |
| 2 | 0 | 0.3535360598259429 | [1221, 779] | 779 | 1221 |
| 2 | 1 | 0.3535360598259429 | [1221, 779] | 779 | 1221 |
| 2 | 2 | 0.3546027095131157 | [1231, 769] | 769 | 1231 |
| 2 | 3 | 0.3535360598259429 | [1221, 779] | 779 | 1221 |
| 2 | 4 | 0.3567097604321432 | [1246, 754] | 754 | 1246 |
| 2 | 5 | 0.3535360598259429 | [1221, 779] | 779 | 1221 |
| 2 | 6 | 0.3540265185976387 | [1226, 774] | 774 | 1226 |
| 2 | 7 | 0.3535360598259429 | [1221, 779] | 779 | 1221 |
| 2 | 8 | 0.3538556660278115 | [1225, 775] | 775 | 1225 |
| 2 | 9 | 0.3535360598259429 | [1221, 779] | 779 | 1221 |
| 3 | 0 | 0.3579632088984379 | [793, 757, 450] | 450 | 793 |
| 3 | 1 | 0.358012583475574 | [793, 758, 449] | 449 | 793 |
| 3 | 2 | 0.3584949957146945 | [804, 765, 431] | 431 | 804 |
| 3 | 3 | 0.3584865120815639 | [803, 763, 434] | 434 | 803 |
| 3 | 4 | 0.3586996370740635 | [809, 760, 431] | 431 | 809 |
| 3 | 5 | 0.3581313150705726 | [794, 758, 448] | 448 | 794 |
| 3 | 6 | 0.3585767176679189 | [805, 760, 435] | 435 | 805 |
| 3 | 7 | 0.3581313150705726 | [794, 758, 448] | 448 | 794 |
| 3 | 8 | 0.3586786660406087 | [805, 757, 438] | 438 | 805 |
| 3 | 9 | 0.3585246111328745 | [806, 760, 434] | 434 | 806 |
| 4 | 0 | 0.3305813998970239 | [740, 540, 382, 338] | 338 | 740 |
| 4 | 1 | 0.3310517269587279 | [734, 537, 372, 357] | 357 | 734 |
| 4 | 2 | 0.3310517269587279 | [734, 537, 372, 357] | 357 | 734 |
| 4 | 3 | 0.3307928122388856 | [731, 537, 380, 352] | 352 | 731 |
| 4 | 4 | 0.3310517269587279 | [734, 537, 372, 357] | 357 | 734 |
| 4 | 5 | 0.3310517269587279 | [734, 537, 372, 357] | 357 | 734 |
| 4 | 6 | 0.3310517269587279 | [734, 537, 372, 357] | 357 | 734 |
| 4 | 7 | 0.3304307680626879 | [740, 538, 381, 341] | 341 | 740 |
| 4 | 8 | 0.3311058250108181 | [733, 537, 372, 358] | 358 | 733 |
| 4 | 9 | 0.3310517269587279 | [734, 537, 372, 357] | 357 | 734 |
| 5 | 0 | 0.3479158741730141 | [648, 399, 394, 369, 190] | 190 | 648 |
| 5 | 1 | 0.3479556361387479 | [648, 401, 391, 369, 191] | 191 | 648 |
| 5 | 2 | 0.347864437909609 | [648, 398, 394, 369, 191] | 191 | 648 |
| 5 | 3 | 0.3479158741730141 | [648, 399, 394, 369, 190] | 190 | 648 |
| 5 | 4 | 0.347864437909609 | [648, 398, 394, 369, 191] | 191 | 648 |
| 5 | 5 | 0.3479158741730141 | [648, 399, 394, 369, 190] | 190 | 648 |
| 5 | 6 | 0.3479158741730141 | [648, 399, 394, 369, 190] | 190 | 648 |
| 5 | 7 | 0.347864437909609 | [648, 398, 394, 369, 191] | 191 | 648 |
| 5 | 8 | 0.3479158741730141 | [648, 399, 394, 369, 190] | 190 | 648 |
| 5 | 9 | 0.3479158741730141 | [648, 399, 394, 369, 190] | 190 | 648 |

## Supplementary Table S22. State variable non-negativity verification.

| **Condition** | **Variable** | **Min_value** | **Max_value** | **Non_negative** |
| --- | --- | --- | --- | --- |
| Standard | A | 2.5568655603184593e-13 | 100.0 | True |
| Standard | Al | 0.0 | 64.78646398757385 | True |
| Standard | DC | 2.799666436455781 | 34.62841039967669 | True |
| Standard | Th2 | 2.0 | 45.22029712936457 | True |
| Standard | Cyto | 0.0 | 512.3459261505142 | True |
| Standard | IgE | 150.0 | 189.91348133576244 | True |
| Standard | MC | 0.0 | 0.8375448172467033 | True |
| Standard | Med | 0.0 | 38.19838331478143 | True |
| Standard | Eos | 9.85493148916846 | 178.9959007253073 | True |
| Standard | Sym | 0.0 | 6.903962714898128 | True |
| Standard | TLR4 | 0.0 | 0.994715489393848 | True |
| Standard | MyD88 | 0.0 | 0.6600561172138474 | True |
| Standard | NFkB | 0.0 | 0.6182137368179065 | True |
| Standard | ProInf | 0.0 | 16.147805650475238 | True |
| Standard | Neu | 4.5705114984779405 | 43.07021991152656 | True |
| Standard | Th17 | 0.9607071130489804 | 12.178039713655249 | True |
| Standard | IL17 | 0.0 | 117.21592123801936 | True |
| Standard | Treg | 4.995168862629438 | 7.708309823595201 | True |
| Standard | IL10 | 2.0000000000000004 | 75.26870967309908 | True |
| High k_Treg_inh (×10) | A | 2.556865742413893e-13 | 100.0 | True |
| High k_Treg_inh (×10) | Al | 0.0 | 64.78646398757226 | True |
| High k_Treg_inh (×10) | DC | 2.739340881075533 | 34.62747824932954 | True |
| High k_Treg_inh (×10) | Th2 | 2.0 | 45.07978738563174 | True |
| High k_Treg_inh (×10) | Cyto | 0.0 | 510.6763631949419 | True |
| High k_Treg_inh (×10) | IgE | 150.0 | 189.72116379322583 | True |
| High k_Treg_inh (×10) | MC | 0.0 | 0.83754481724427 | True |
| High k_Treg_inh (×10) | Med | 0.0 | 38.19838330997632 | True |
| High k_Treg_inh (×10) | Eos | 9.854931446889298 | 178.9334541714904 | True |
| High k_Treg_inh (×10) | Sym | 0.0 | 6.903824747455224 | True |
| High k_Treg_inh (×10) | TLR4 | 0.0 | 0.994715489393848 | True |
| High k_Treg_inh (×10) | MyD88 | 0.0 | 0.6600561172138901 | True |
| High k_Treg_inh (×10) | NFkB | 0.0 | 0.6182137368179695 | True |
| High k_Treg_inh (×10) | ProInf | 0.0 | 16.14780565047444 | True |
| High k_Treg_inh (×10) | Neu | 4.557154853793166 | 34.50805752216843 | True |
| High k_Treg_inh (×10) | Th17 | -1.852450386480844 | 8.497145941028935 | False |
| High k_Treg_inh (×10) | IL17 | -4.205173016224387 | 79.36140686562187 | False |
| High k_Treg_inh (×10) | Treg | 4.995238756831836 | 7.863031539781877 | True |
| High k_Treg_inh (×10) | IL10 | 2.0000000000000004 | 77.02588486633502 | True |
| High k_Th17_inh (×10) | A | 2.556865039887019e-13 | 100.0 | True |
| High k_Th17_inh (×10) | Al | 0.0 | 64.78646398757317 | True |
| High k_Th17_inh (×10) | DC | 4.673602399259064 | 34.651959302485224 | True |
| High k_Th17_inh (×10) | Th2 | 2.0 | 45.6503777179251 | True |
| High k_Th17_inh (×10) | Cyto | 0.0 | 517.654983432183 | True |
| High k_Th17_inh (×10) | IgE | 150.0 | 190.5801472296255 | True |
| High k_Th17_inh (×10) | MC | 0.0 | 0.8375448172466984 | True |
| High k_Th17_inh (×10) | Med | 0.0 | 38.19838331484521 | True |
| High k_Th17_inh (×10) | Eos | 9.854931489237169 | 179.1936199559018 | True |
| High k_Th17_inh (×10) | Sym | 0.0 | 6.90458027166303 | True |
| High k_Th17_inh (×10) | TLR4 | 0.0 | 0.994715489393848 | True |
| High k_Th17_inh (×10) | MyD88 | 0.0 | 0.6600561172138762 | True |
| High k_Th17_inh (×10) | NFkB | 0.0 | 0.6182137368179348 | True |
| High k_Th17_inh (×10) | ProInf | 0.0 | 16.14780565047483 | True |
| High k_Th17_inh (×10) | Neu | 4.570514029632273 | 43.422435500798215 | True |
| High k_Th17_inh (×10) | Th17 | 0.9607856598432952 | 12.337981222031653 | True |
| High k_Th17_inh (×10) | IL17 | 0.0 | 119.02219997698955 | True |
| High k_Th17_inh (×10) | Treg | 0.1410577908734046 | 5.751582958946986 | True |
| High k_Th17_inh (×10) | IL10 | 2.0000000000000004 | 51.44502080494549 | True |
| High k_S_IL10 (×10) | A | 2.5568367344336405e-13 | 100.0 | True |
| High k_S_IL10 (×10) | Al | 0.0 | 64.7864640514501 | True |
| High k_S_IL10 (×10) | DC | 2.7996664212438658 | 34.628410390680685 | True |

... (92 more rows)

## Supplementary Figure S1

H4 Directional Consistency Assessment: (A) Th17/Treg ratio vs late-phase AUC correlation. (B) IgE vs late-phase AUC. (C) Tertile comparison. (D) ROC curves. See main text Figure 12 and Supplementary Table S16 for data.
